# Comparative Antennal Morphology of *Agriotes* (Coleoptera: Elateridae), with Special Reference to the Typology and Possible Functions of Sensilla

**DOI:** 10.3390/insects11020137

**Published:** 2020-02-21

**Authors:** Michel J. Faucheux, Tamás Németh, Robin Kundrata

**Affiliations:** 1 Laboratoire d’Endocrinologie des Insectes Sociaux, Faculté des Sciences et des Techniques, 2 rue de la Houssinière, B.P. 92208, F-44322 Nantes CEDEX 03, France; faucheux.michel@free.fr; 2 Department of Zoology, Hungarian Natural History Museum, Baross utca 13, H-1088 Budapest, Hungary; nemeth.tamas@nhmus.hu; 3 Department of Zoology, Faculty of Science, Palacky University, 17. listopadu 50, CZ-771 46 Olomouc, Czech Republic

**Keywords:** click-beetles, chemoreceptors, crop pest, pheromones, pest control, scanning electron microscopy, wireworm

## Abstract

Species of the click-beetle genus *Agriotes* Eschscholtz are economically important crop pests distributed mainly in the Northern Hemisphere. They can inflict considerable damage on various field crops. Therefore, the detection, monitoring, and control of *Agriotes* include the adult trapping using species-specific sex pheromones, which is a critical component of pest research. To obtain a better understanding of the detailed antennal morphology as background information for subsequent chemical ecology research, we conducted a scanning electron microscopy study of the antennal sensilla of both sexes in 10 European *Agriotes* species. We identified 16 different sensilla in *Agriotes*, belonging to six main types: sensilla chaetica (subtypes C1 and C2), sensilla trichodea, sensilla basiconica (subtypes B1–B9), dome-shaped sensilla (subtypes D1 and D2), sensilla campaniformia, and Böhm sensilla. We discuss their possible functions and compare the sensilla of *Agriotes* with those of other Elateridae in order to consolidate the sensillum nomenclature in this family. Additionally, our study reveals the remarkable interspecific variability in sensillar equipment of *Agriotes* and identifies several characters of potential importance for future use in systematic studies. The present study provides a strong preliminary framework for subsequent research on the antennal morphology of this crop pest on a wider scale.

## 1. Introduction

The click-beetles of genus *Agriotes* Eschscholtz, 1829 (Elateridae: Elaterinae: Agriotini) are among the most serious agricultural pests. Their larvae, wireworms, are abundant soil-dwelling insects that can cause considerable damage to a wide variety of crops such as potato, cereals, sugar beet, vegetables, or fruit [1,2,3,4]. They significantly reduce yields or crop value by primary damage of various plant parts, which can subsequently facilitate secondary crop damage by pathogens [5,6]. The genus *Agriotes* is distributed mainly in the Northern Hemisphere; approximately 150 species are known from the Palaearctic region and 40 from the Nearctic region [7,8,9]. In Europe, nine species are recognized as pests of special agricultural importance, i.e., *Agriotes brevis* Candèze, 1863, *A. lineatus* (Linnaeus, 1767), *A. litigiosus* (Rossi, 1792), *A. obscurus* (Linnaeus, 1758), *A. proximus* Schwarz, 1891, *A. rufipalpis* Brullé, 1832, *A. sordidus* (Illiger, 1807), *A. sputator* (Linnaeus, 1758), and *A. ustulatus* (Schaller, 1783) [4,10]. Numerous papers have been published on the economically important *Agriotes* species to date [6]. Although recent research has significantly increased our understanding of the biology, ecology, life-cycle, and morphology of several species, more comprehensive studies including many more species from various zoogeographical regions are needed [3,6]. Moreover, the taxonomy of *Agriotes* should be revised in detail as suggested by molecular phylogenetic analyses and pheromone composition [11,12,13]. The morphological determination of some species is still debatable, which precludes subsequent applied research [4,14].

The pest control and management of the *Agriotes* click-beetles are crucial for reducing crop damage. This includes not only soil sampling and bait systems for wireworms but also adult trapping using pheromones [2,3]. Increased knowledge of sex-specific pheromones of *Agriotes* species has led to the development of several species-specific pheromone traps that seem to be an effective tool for the detection, monitoring, and control of these pests [15,16,17,18,19]. Therefore, it is important to understand sexual communication in *Agriotes* in detail. While there is a growing number of papers dealing with sex pheromone gland morphology and mainly the pheromone components [13,20,21,22,23,24,25], our knowledge of chemoreception and sensory systems of *Agriotes* antennae is still limited. Antennal sensilla of insects play a role in the recognition of sex pheromones, so it is important to study their structure and distribution. In *Agriotes*, the antennal sensillar equipment was studied only for *A. obscurus* [26,27]. The authors focused mainly on the distribution of olfactory sensilla. However, a more detailed, comparative study including both sexes of additional *Agriotes* species is needed to better understand the antennal sensory equipment of these pests.

In this study, we examined male and female antennal morphology of 10 *Agriotes* species including five that are among the most significant European pest species [10]. Our main goals were to identify the types of antennal sensilla in *Agriotes*, discuss their possible functions, compare the inter- and intraspecific variability in morphology, number, and distribution of antennal sensilla, and compare the sensillar equipment of *Agriotes* with other Elateridae [28,29,30,31,32,33].

## 2. Materials and Methods

**Figure 1:**
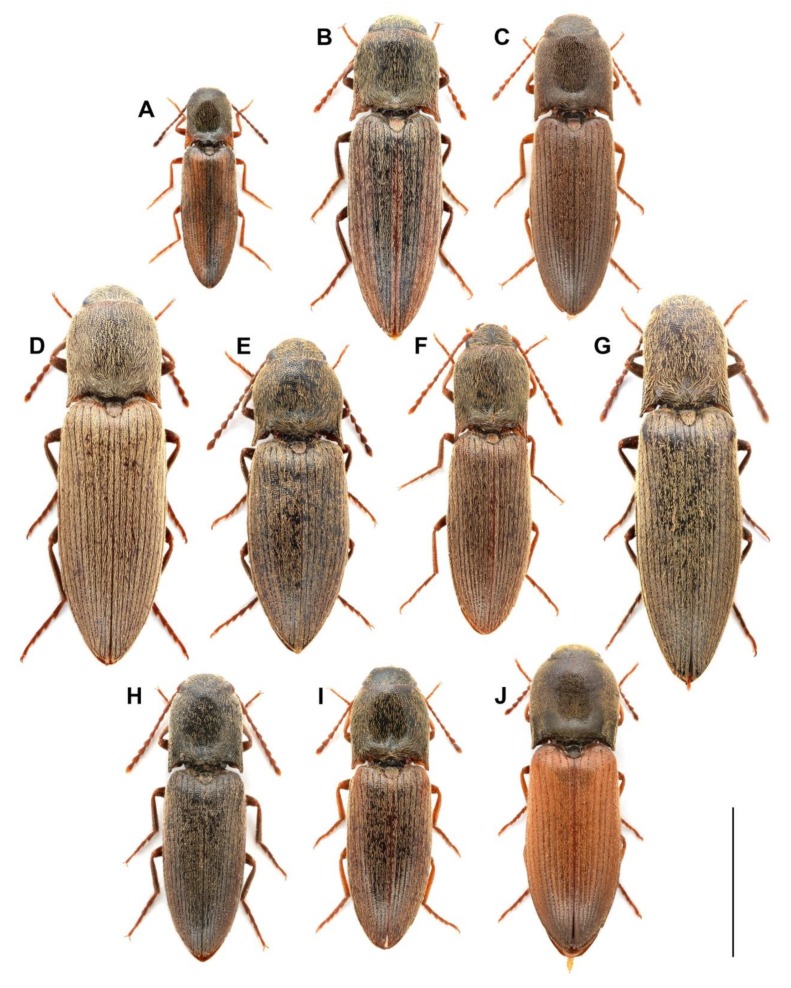
Habitus images of the examined *Agriotes* species. (**A**) *A. acuminatus*; (**B**) *A. lineatus*; (**C**) *A. medvedevi*; (**D**) *A. modestus*; (**E**) *A. obscurus*; (**F**) *A. paludum*; (**G**) *A. pilosellus*; (**H**) *A. rufipalpis*; (**I**) *A. sputator*; (**J**) *A. ustulatus*. Scale bar = 5 mm.

We examined the morphology, number, and distribution of the sensilla in both male and female antennae of 10 European *Agriotes* species (42 adult specimens in total), i.e., *A. acuminatus* (Stephens, 1830), *A. lineatus*, *A. medvedevi* Dolin, 1960, *A. modestus* Kiesenwetter, 1858, *A. obscurus*, *A. paludum* Kiesenwetter, 1858, *A, pilosellus* (Schönherr, 1817), *A. rufipalpis*, *A. sputator*, and *A. ustulatus*. All species except *A. medvedevi*, *A. modestus,* and *A. sputator* were represented by specimens from different populations. The geographic origin of the examined material is given in Table 1, and habitus images of all species are included in Figure 1. The specimens are deposited in the collection of the first author (Nantes, France).

For the scanning electron microscopy (SEM) study, the antennae were either removed from the head or not, cleaned in acetone, dehydrated in pure ethanol, air-dried, and mounted both on the dorsal and the ventral face on specimen holders. After coating with gold and palladium in a vacuum evaporator, antennae were examined using a Jeol J.S.M. 6 400F scanning electron microscope at 10 kV. The numbers of each sensillum type were calculated based on the examination of 11 antennomeres of both the left and right antenna with the SEM. The sensilla were classified according to their external morphology, distribution, and presence or absence of pores following Zacharuk [34], Altner and Prillinger [35], Faucheux [36], and partly also Merivee [26] (for *Agriotes obscurus* (Linnaeus, 1758).

## 3. Results

### 3.1. Gross Morphology of Antennae in Agriotes Species

**Figure 2:**
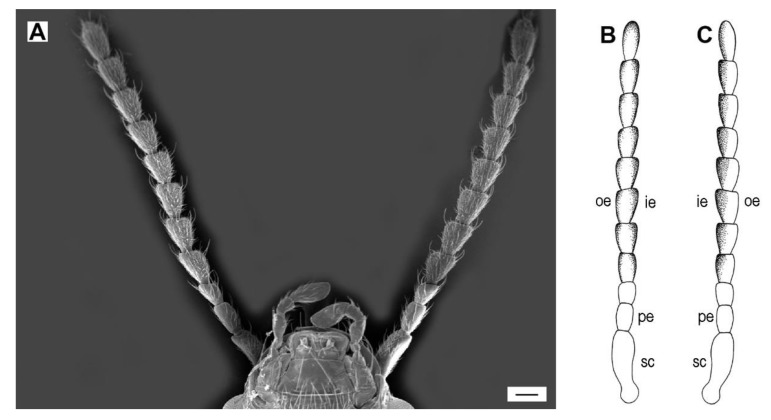
General morphology of *Agriotes* antennae. (**A**) *A. acuminatus*, male, antennae and frontal part of head, ventral view; (**B**) Sensillar fields on dorsal surface of antennae, schematic; (**C**) Sensillar fields on ventral surface of antennae, schematic. oe, outer edge; ie, inner edge; sc, scape; pe, pedicel. Scale bar = (**A**): 100 μm; (**B**,**C**): not to scale.

**Figure 3:**
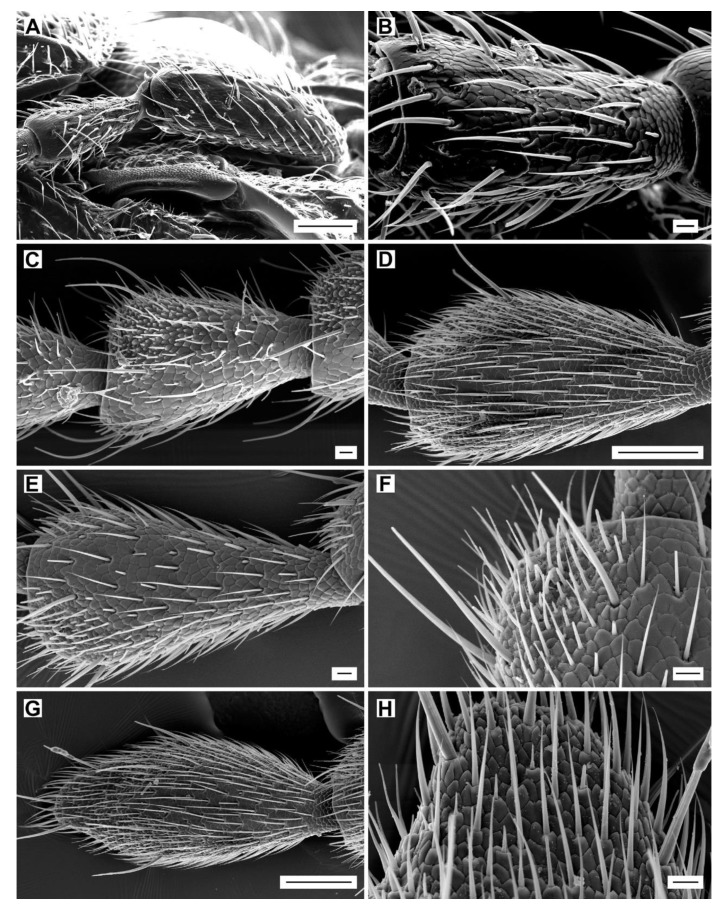
Surface of antennomeres and location of the sensillar fields. (**A**) *A. medvedevi*, male, antennomeres I and II, ventrally; (**B**) *A. medvedevi*, male, antennomere III, ventrally; (**C)** *A. acuminatus*, male, antennomeres IV–VI, ventrally; (**D**) *A. modestus*, male, antennomere IX, dorsally; (**E**) *A. medvedevi*, male, antennomere X, dorsally; (**F**) dtto, detail; (**G**) *A. modestus*, male, antennomere XI, dorsally; (**H**) dtto, detail. Scale bars = (**A**,**D**,**G**): 100 μm; (**B**,**C**,**E**,**F**,**H**): 10 μm.

The antennae of the examined *Agriotes* species are moderately long and slightly serrate (Figure 2A–C). No significant differences in general shape and structure of antenna were found between males and females. The gross antennal morphology consists of the scape (antennomere I), the pedicel (antennomere II), and the flagellum that is composed of nine flattened flagellomeres (antennomeres III–XI) (Figure 2A–C). The scape (Figure 3A) is the longest antennomere, somewhat curved and concave on the inner side, sub-basally constricted, and basally articulated with an antennal fossa by a globular condyle, which enables antennal movement. The pedicel (Figure 3B) is elongate, basally articulated with the scape by a small globular condyle. Antennomere III (i.e., flagellomere I) is the shortest antennomere, only slightly longer than wide. Each of the antennomeres IV–X is somewhat pear-shaped, narrower basally, gradually widened toward the apex, apically about twice as wide as in the basal part, with the inner edge slightly more expanded (Figure 2A–C and Figure 3C–F). Most sensilla are located within the inner and outer sensillar fields on the inner and outer edges of each antennomere, respectively. The inner sensillar field is much larger and is visible on both the ventral (larger part) and dorsal (smaller part) faces. The outer sensillar field is smaller and is usually visible only on the dorsal face (Figure 2B,C). The microsculpture of the sensillar fields is formed by small, bulged cuticular scales, while the microsculpture outside the sensillar fields consists of larger, more or less flat cuticular scales (Figure 3C–H). Antennomere XI is elongate-oval, with the inner and outer sensillar fields fused together (Figure 2B and Figure 3G,H). The ratios of antennomere lengths and the total antennal lengths for both sexes of all examined species are given in Table S1.

### 3.2. Types of Sensilla in Agriotes Species

Six types of sensilla were identified on the antennae of *Agriotes* species: sensilla chaetica (subtypes C1 and C2), sensilla trichodea, sensilla basiconica (subtypes B1–B9), dome-shaped sensilla (subtypes D1 and D2), sensilla campaniformia, and Böhm sensilla. Glandular pores corresponding to the integumental glands were also present. The morphological characteristics of different types and subtypes of antennal sensilla in the examined *Agriotes* species are summarized in Table 2. Distribution of the sensillum types and subtypes on different antennomeres of the examined *Agriotes* species is summarized in Table 3. Percentages of different types of sensilla for all specimens are listed in Table 4. Numbers of types and subtypes of sensilla on individual antennomeres for all species are given in Tables S2–S11.

#### 3.2.1. Sensilla chaetica (subtypes C1–C2)

**Figure 4:**
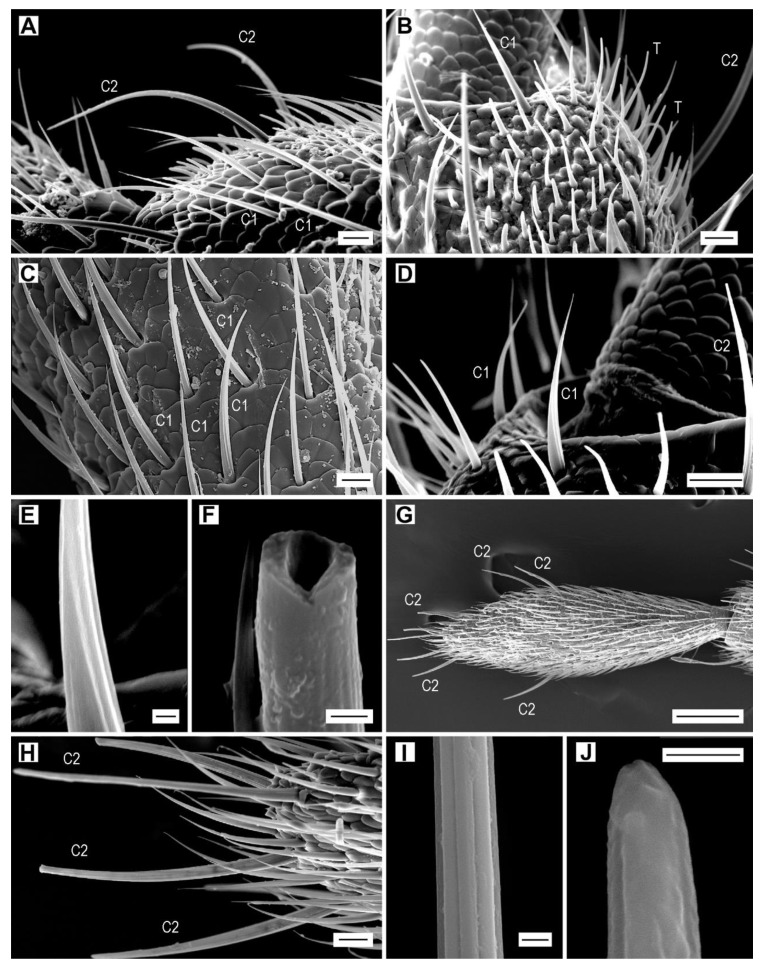
Sensilla chaetica. (**A**) *A. acuminatus*, male, numerous sensilla chaetica C1 and C2 on antennomere III; (**B**) *A. lineatus*, male, sensilla chaetica and trichodea; (**C**) *A. obscurus*, male, sensilla chaetica C1; (**D**) *A. medvedevi*, male, sensilla chaetica C1 and C2; (**E**) *A. medvedevi*, male, detail of the wall of sensillum C1; (**F**) *A. pilosellus*, male, broken sensillum C1; (**G**) *A. modestus*, female, antennomere XI, dorsally, sensilla chaetica C1 and C2; (**H**) dtto, detail; (**I**) *A. modestus*, female, striae on the wall of sensillum C2; (**J**) *A. obscurus*, male, apex of sensillum C2. Scale bars = (**A**–**D**,**H**): 10 μm; (**E**,**F**,**I**,**J**): 1 μm; (**G**): 100 μm.

Sensilla chaetica C1 are long, sickle-shaped, nonporous hairs with a widened base, gradually tapering apex, thick wall with a reduced lumen (clearly visible on broken sensilla), and usually 15 more or less apparent striae that meet distally, with two neighboring forming a letter “V” (Figure 4A–F). These sensilla are inserted in a wide cuticular socket and are movable at their base. Most of them are semi-erect and bent toward the apex of antenna so that their apical portions are subparallel to the antennomere surface (Figure 4A). Sensilla C1 are the only sensilla with a wide length range (30–110 µm), although the majority are approximately 70–80 µm long (Table 2). Their length usually increases from the proximal to the distal region of the antennomere, with a decrease in length on the distal edge. For example, on antennomere V of *A. acuminatus,* sensilla C1 increase from 45 µm in the proximal part to 61 µm in the distal part, and the distal edge bears only 32-µm-long sensilla C1. The longest sensilla C1 are usually found on the scape (e.g., up to 85 µm in *A. acuminatus*), then on antennomeres III to VIII they decrease in maximum length, in antennomeres IX and X they are again longer, and they are usually quite short on the apical antennomere, on which they are clearly shorter than the sensilla chaetica C2 (Figure 4G). Sensilla C1 are evenly distributed over the surface of each antennomere except for the sensillar fields where they are rare. They are usually least numerous on antennomeres II and III and most numerous on the apical antennomere (except for males of *A. pilosellus* and *A. acuminatus*, in which sensilla C1 are most numerous on the scape) (Tables S2–S11). Sensilla C1 are usually the most frequent sensilla in all *Agriotes* species, comprising up to 65.3% of all sensilla (˃1000 sensilla per antenna in all species except for *A. acuminatus* and *A. ustulatus*). The only exception is *A. acuminatus*, which has more sensilla basiconica than sensilla chaetica. Sensilla C1 display sexual dimorphism; they are more abundant in females (Table 3).

Sensilla chaetica C2 are long, curved, and erect, usually forming a right angle with the cuticle. They are parallel-sided in the basal half and then gradually narrowed toward apex (Figure 4A,D,G,H). The wall of sensillum C2 possesses eight shallow striae (Figure 4I); the apex is blunt and with a barely visible terminal pore, which is surrounded by an uneven surface (Figure 4J). Sensilla C2 are arranged in a circle around the distal part of each antennomere, except for the apical antennomere that has a median circle and an apical circle (Figure 4G). Their number per antennomere is relatively small and varies from 3 to 16 depending on the species and antennomere, with the lowest numbers on the first three antennomeres and the highest numbers on the apical antennomere. The total number of sensilla C2 per antenna is always higher in females (70–88) than in males (60–79). *Agriotes pilosellus* has the least number of sensilla C2 in both sexes, while *A. ustulatus* and *A. paludum* have the highest numbers of these sensilla (Tables S2–S11). The sensilla C2 form 2.0–4.3% of the total antennal sensilla in males and 2.3–4.6% in females.

#### 3.2.2. Sensilla trichodea

**Figure 5:**
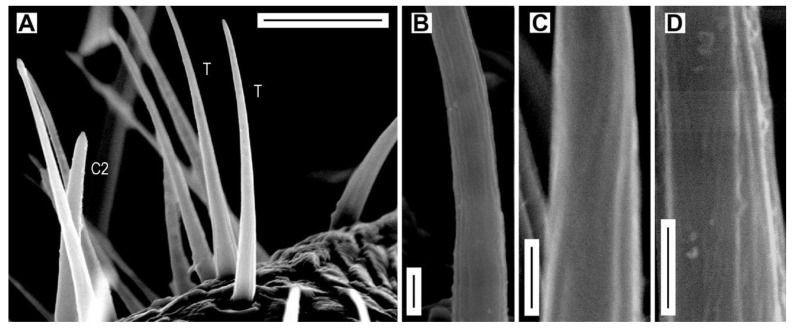
Sensilla trichodea. (**A**) *A. medvedevi*, male, sensilla trichodea (T) and sensillum chaeticum C2; (**B**) *A. acuminatus*, female, detail of wall of sensillum trichodeum; (**C**) *A. medvedevi*, male, detail of wall of sensillum trichodeum; (**D**) *A. lineatus*, male, detail of wall of sensillum trichodeum. Scale bars = (**A**): 10 μm; (**B**–**D**): 1 μm.

Sensilla trichodea are superficially similar to sensilla chaetica C1 but they differ in the shorter length (Figure 4B and Figure 5A), narrower basal diameter (Table 2), and the erect position. They lack the cuticular collar at the base. The wall of sensillum trichodeum includes 20 longitudinal striae, which are apparently shallower than those on sensilla chaetica C1 and C2 (Figure 5B,C), and there are presumable rows of wall pores (Figure 5D).

Sensilla trichodea are present only on antennomeres IV–XI, and their number per antennomere is gradually increasing toward the apex, especially in males (Table 3, Tables S2–S11). Sensilla trichodea are located in groups and only within the distal sensillar fields. There is strong sexual dimorphism in the number of sensilla trichodea per antenna in all *Agriotes* species; it varies from 394 (*A. acuminatus*) to 626 (*A. obscurus*) in males and from 73 (*A. medvedevi*) to 155 (*A. modestus*) in females, and males have 3.1 (*A. modestus*) to 6.5 times (*A. lineatus*) more sensilla trichodea per antenna than females. The proportion of sensilla trichodea from the total number of sensilla per antenna varies between 13.9% (*A. modestus*) to 26.5% (*A. acuminatus*) in males and between 2.9% (*A. medvedevi*) to 7.3% (*A. ustulatus*) in females. There is only small variability in the number and proportion of sensilla trichodea within the examined *Agriotes* species and populations (Table 4).

#### 3.2.3. Sensilla basiconica (subtypes B1–B9)

Sensilla basiconica in the examined *Agriotes* species are divided into seven subtypes (B1–B7) that are present in all species and two subtypes (B8, B9) that can be found only in one or two species (Table 3). They are the most numerous antennal sensilla in *A. acuminatus* and the second most numerous in the remaining *Agriotes* species. Sensilla basiconica are relatively more abundant in females. They form 18.6–38.4% of the total sensilla in males and 23.3–43.2% in females (Table 4).

**Figure 6:**
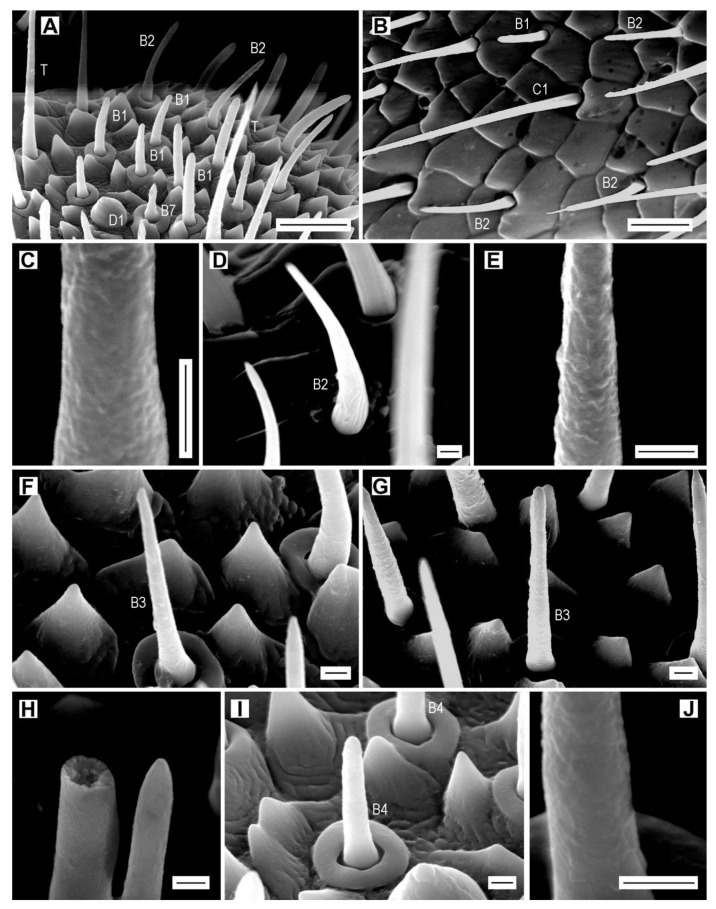
Sensilla basiconica of subtypes B1–B4. (**A**) *A. acuminatus*, male, dome-shaped sensillum D1, sensilla trichodea (T) and sensilla basiconica B1, B2, and B7; (**B**) *A. acuminatus*, female, sensilla basiconica B1 and B2, and sensilla chaetica C1; (**C**) *A. acuminatus*, male, wall pores of sensillum B1; (**D**) *A. medvedevi*, male, sensillum basiconicum B2; (**E**) *A. acuminatus*, male, detail of the wall of sensillum B2; (**F**) *A. acuminatus*, male, sensillum basiconicum B3; (**G**) *A. acuminatus*, male, sensillum basiconicum B3; (**H**) *A. pilosellus*, female, sensilla basiconica B3, left: broken, right: apical part; (**I**) *A. acuminatus*, male, sensilla basiconica B4; (**J**) *A. acuminatus*, male, detail of the wall of sensillum B4. Scale bars = (**A**,**B**): 10 μm; (**C**–**J**): 1 μm.

Sensilla B1 and B2 form the vast majority of total sensilla basiconica in *Agriotes* (Tables S2–S11). They are usually the longest sensilla basiconica subtypes (Table 2). They are located in the sensillar fields on antennomeres IV–XI of all species and their number per antennomere usually increases toward the apex of the antenna (Table 3, Tables S2–S11). Both subtypes are more abundant in females. They are elongated pegs with blunt apical parts, wall pores, and a cuticular collar, which is usually 5–7 µm wide (Figure 6A–E). Compared to B1, subtype B2 is relatively narrower (especially in apical half) and distally curved (Figure 6D). The total number of sensilla B1 per antenna varies between 333 (*A. modestus*) and 548 (*A. pilosellus*) in males and between 417 (*A. ustulatus*) and 642 (*A. pilosellus*) in females. The total number of sensilla B2 per antenna varies between 178 (*A. acuminatus*) and 338 (*A. pilosellus*) in males and between 183 (*A. acuminatus*) and 375 (*A. pilosellus*) in females (Tables S2–S11).

Sensilla B3 resemble B1 and B2 but they are narrower along their whole length (Figure 6F–H, Table 2). They have a relatively thin wall with pores (Figure 6H). These sensilla are usually located on antennomeres X and XI, but those on antennomere X are missing in both sexes of *A. lineatus* and males of *A. medvedevi*, *A. paludum*, and *A. pilosellus* (Table 3, Tables S2–S11). The total number of sensilla B3 per antenna varies between four (*A. pilosellus*) and eight (*A. modestus*, *A. ustulatus*) in males and between nine (*A. lineatus*) and 13 (*A. medvedevi*) in females. Females always have more sensilla B3 than males, in some species only slightly more, e.g., *A. lineatus* (Table S3), and in other species more than twice as many, e.g., *A. medvedevi* (Table S4).

Subtype B4 is a relatively short and narrow peg with a large cuticular collar (usually 4.7 µm in outer diameter) and a rounded apex (Figure 6I, Table 2). The wall pores are less visible than those of the previous basiconica subtypes but the pimply surface of the peg clearly suggests their presence (Figure 6J). These sensilla are scattered among the clusters of sensilla B1 in the sensillar fields. They are present in small numbers (always less than 10) on antennomeres VII–XI (all females, and males of *A. lineatus*, *A. obscurus*, *A. paludum*, and *A. pilosellus*) or VIII–XI (males of *A. acuminatus*, *A. medvedevi*, *A. modestus*, *A. rufipalpis*, *A. sputator*, and *A. ustulatus*) (Table 3, Tables S2–S11). The total number of sensilla B4 per antenna varies between 16 (*A. lineatus*, *A. pilosellus*) and 20 (*A. modestus*) in males, and between 22 (*A. modestus*, *A. pilosellus*, *A. ustulatus*) and 26 (*A. paludum, A. rufipalpis*) in females.

**Figure 7:**
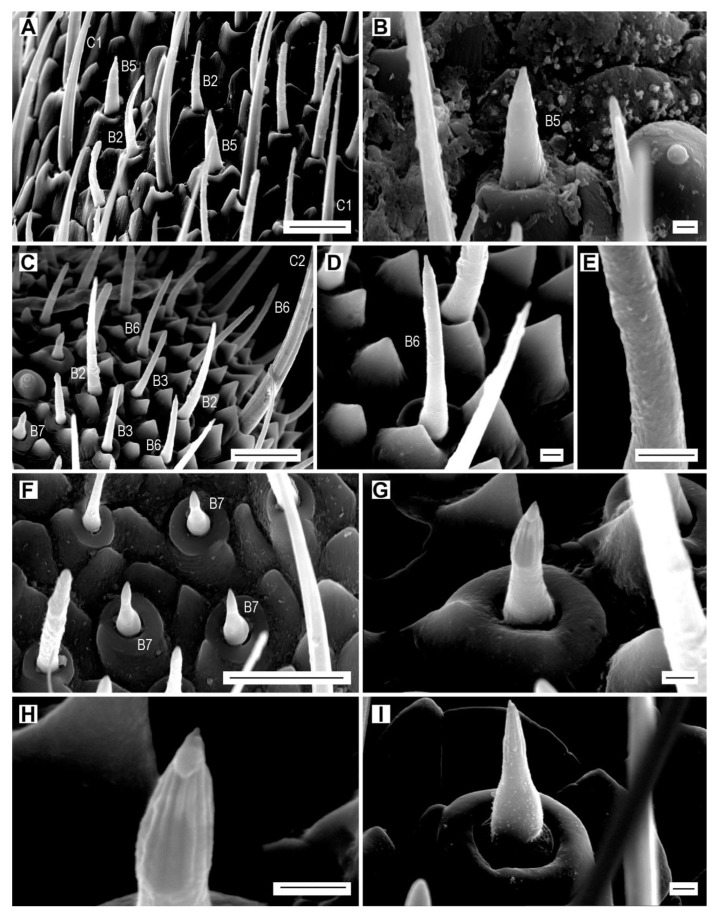
Sensilla basiconica of subtypes B5–B7. (**A**) *A. modestus*, female, sensilla chaetica C1 and basiconica B2 and B5 on antennomere XI; (**B**) *A. pilosellus*, male, sensillum basiconicum B5; (**C**) *A. acuminatus*, male, sensilla basiconica B2, B3, B6, and B7, and sensillum chaeticum C2; (**D**) *A. acuminatus*, male, sensillum basiconicum B6; (**E**) detail of the wall of sensillum B6; (**F**) *A. rufipalpis*, female, sensilla basiconica B7; (**G**) *A. acuminatus*, male, sensillum basiconicum B7; (**H**) detail of sensillum B7 showing the striae; (**I**) *A. obscurus*, female, sensillum basiconicum B7. Scale bars = (**A**,**C**,**F**): 10 μm; (**B**,**D**,**E**,**G**–**I**): 1 μm.

Sensilla basiconica B5 are short stout cones with a characteristic terminal nipple (Figure 7A,B, Table 2). The wall pores are not well visible but their presence is hypothesized. They are usually located in groups of two near the dome-shaped sensilla D1 within the sensillar fields on antennomeres X and XI, but those on antennomere X are missing in both sexes of *A. acuminatus* and *A. paludum*, and females of *A. medvedevi* and *A. sputator* (Figure 7B, Table 3, Tables S2–S11). Males always have more sensilla B5 than females, and in species with sensilla B5 on the two apical antennomeres, the apical one always bears more sensilla. The total number of sensilla B5 per antenna varies between four (*A. paludum*) and nine (*A. pilosellus*, *A. ustulatus*) in males and between three (*A. acuminatus*, *A. paludum*) and seven (*A. pilosellus*) in females.

Sensilla basiconica B6 resemble sensilla B5 but they are approximately twice as long, have an abruptly tapering tip, and well visible wall pores (Figure 7C–E, Table 2). These sensilla are present only in small numbers (2–7) on the apical antennomere in all species and they are usually slightly more abundant in males (Table 3, Tables S2–S11).

Subtype B7 is a relatively short peg with a more or less large bulbous base that is abruptly narrowing toward apex, forming a sharp-tipped cone (Figure 7F–I). The base of the peg is located in a deeply and irregularly striate convex pedestal that is surrounded by a cuticular bulge that has its outer diameter wider than the length of the sensillum. There are 14–16 longitudinal striae with the wall pores on the distal half of the peg (Figure 7G,H). In *A. obscurus*, the peg is more elongated and the grooves are less numerous (Figure 7I). Sensilla B7 usually form groups within the sensillar fields on antennomeres IV–XI (Figure 7F). They are most numerous on the apical antennomere. These sensilla are absent on antennomere V in females of *A. acuminatus*, on antennomere VI in females of *A. sputator*, and on antennomere VII in males of *A. medvedevi* (Table 3, Tables S2–S11). In most *Agriotes* species (except *A. acuminatus* and *A. sputator*), males have more sensilla B7 than females. The total number of sensilla B7 per antenna varies between 21 (*A. ustulatus*) and 34 (*A. rufipalpis*) in males and between 23 (*A. sputator*) and 41 (*A. rufipalpis*) in females (Table 3, Tables S2–S11).

**Figure 8:**
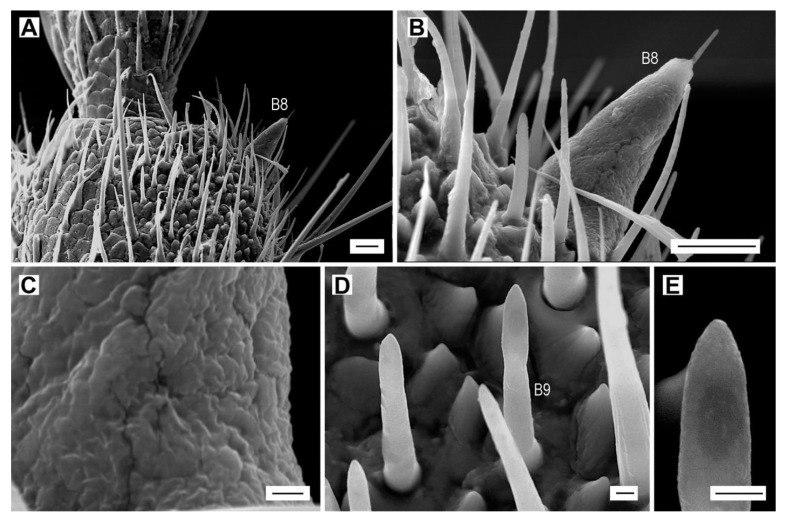
Sensilla basiconica of subtypes B8 and B9. (**A**) *A. lineatus*, male from Romania, sensillum basiconicum B8 on the distal part of antennomere X; (**B**) sensillum basiconicum B8; (**C**) detail of the wall of sensillum B8; (**D**) *A. pilosellus*, female, sensillum basiconicum B9 on antennomere VIII; (**E**) detail of the apex of sensillum B9. Scale bars = (**A**,**B**): 10 μm; (**C**–**E**): 1 μm.

Sensillum basiconicum B8 is a stout, long, triangular peg with a rugged surface (Figure 8A–C, Table 2). We found it only in a single male of *A. lineatus* from Romania. A single sensillum is located along the distal part of the outer edge on antennomere X (Figure 8A).

Sensilla B9 are tongue-shaped, with an enlarged base and barely visible wall-pores (Figure 8D,E). They are present only in both sexes of *A. pilosellus*. Six sensilla in the males and seven in the females are distributed on antennomeres IX–XI (Table 3, Table S8).

#### 3.2.4. Dome-shaped Sensilla (subtypes D1–D2)

**Figure 9:**
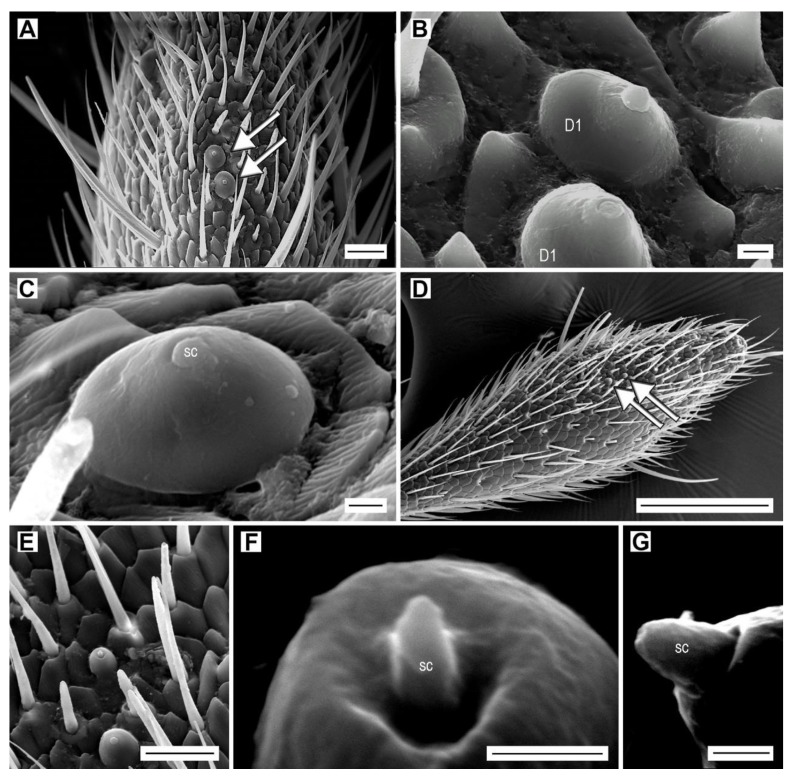
Dome-shaped sensilla. (**A**) *A. acuminatus*, male, location of dome-shaped sensilla D1 (arrows) on antennomere XI; (**B**) detail of dome-shaped sensilla D1; (**C**) *A. rufipalpis*, female, dome-shaped sensillum D1 showing the sensory cone (sc); (**D**) *A. medvedevi*, male, location of dome-shaped sensilla D2 (arrows) on antennomere XI; (**E**) *A. medvedevi*, male, two dome-shaped sensilla D2; (**F**) *A. medvedevi*, male, detail of the sensory cone of sensillum D2; (**G**) *A. acuminatus*, male, detail of the sensory cone of sensillum D2. Scale bars = (**A**,**E**): 10 μm; (**B**,**C**,**F**,**G**): 1 μm; (**D**) = 100 μm.

The dome-shaped sensilla of subtype D1 are composed of a short, globular sensory cone (approximately 1 µm long and 1.25 µm wide basally) with a blunt apex, located apically on a dome, which is clearly wider than long (Figure 9A–C). They are located only in the sensillar fields of antennomeres IV–XI and often in a group of two or three sensilla (Figure 9A). Antennomeres IV–X each bear one to four sensilla, and the apical antennomere bears 8–13 sensilla (Tables S2–S11). The total number of sensilla D1 per antenna varies between 20 (*A. acuminatus*, *A. medvedevi*, *A. sputator*) and 30 (*A. obscurus*) in males and between 19 (*A. pilosellus*) and 32 (*A. obscurus*) in females (Table 3, Tables S2–S11).

Dome-shaped sensilla D2 are present only in *A. acuminatus* and *A. medvedevi* (Table 3, Tables S2 and S4). In both sexes, two sensilla are located on the apical antennomere (Figure 9D). Sensillum D2 has a smaller dome and a longer sensory cone than D1 (Table 2). In *A. medvedevi*, the sensory cone is 1.4 µm long and 0.6 µm wide basally, and it is sunken into an apical cavity of the dome (Figure 9E,F). In *A. acuminatus*, the cone is 1.3 µm long and 0.8 µm wide basally (Figure 9G). The surface of these sensilla is not smooth and most probably contains wall pores (Figure 9F,G).

#### 3.2.5. Sensilla campaniformia

**Figure 10:**
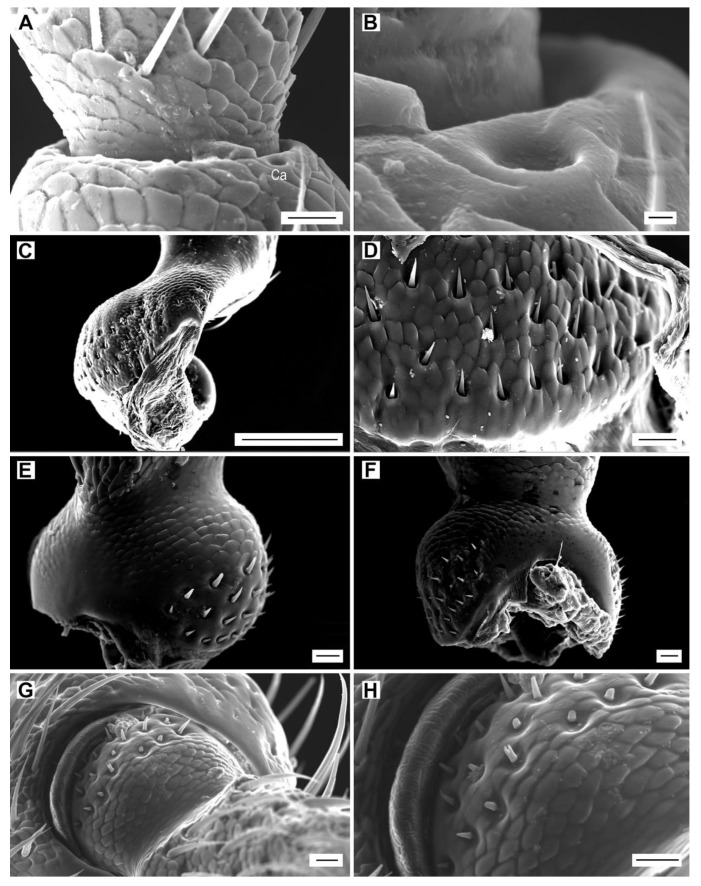
Sensilla campaniformia and Böhm sensilla. (**A**) *A. acuminatus*, male, location of campaniform sensillum (Ca) on the distal part of pedicel; (**B**) detail of campaniform sensillum; (**C**) *A. modestus*, male, Böhm sensilla on the scape; (**D**) detail of Böhm sensilla on the scape; (**E**) *A. lineatus*, male, Böhm sensilla on the pedicel, dorsally; (**F**) *A. pilosellus*, male, Böhm sensilla on the pedicel, ventrally; (**G**) *A. obscurus*, female, Böhm sensilla on the pedicel, dorsally (**H**) detail of the dorsal region of pedicel with Böhm sensilla. Scale bars = (**A**,**D**–**G**): 10 μm; (**B**): 0.1 μm; (**C**): 100 μm; (**H**): not to scale.

These sensilla have a small circular cuticular cap, approximately 3 µm in diameter, embedded in the integument and surrounded by a ring of raised cuticle with an inner diameter of 3.3 µm and an outer diameter of 6.2 µm (Figure 10A,B). In each of the examined *Agriotes* specimens, a single campaniform sensillum is located at the distal edge of the pedicel, near the joint with antennomere III, usually on the ventral face (Figure 10A).

#### 3.2.6. Böhm sensilla

Böhm sensilla are short, smooth, sharp-tipped, thorn-like bristles (Figure 10C–H). In all species, they are located in three clusters at the base (condyle) of the scape (Figure 10C,D) and in two clusters at the base of the pedicel (Figure 10E–H). Within a given cluster, their length can vary from 3.3 to 9.2 µm. Several sensilla per specimen have a bifid tip (Figure 10G,H). There are no significant differences in the number of Böhm sensilla among *Agriotes* species, sexes and geographically distant populations of a single species (112–117 sensilla per antenna) (Tables S2–S11).

#### 3.2.7. Glandular pores

**Figure 11:**
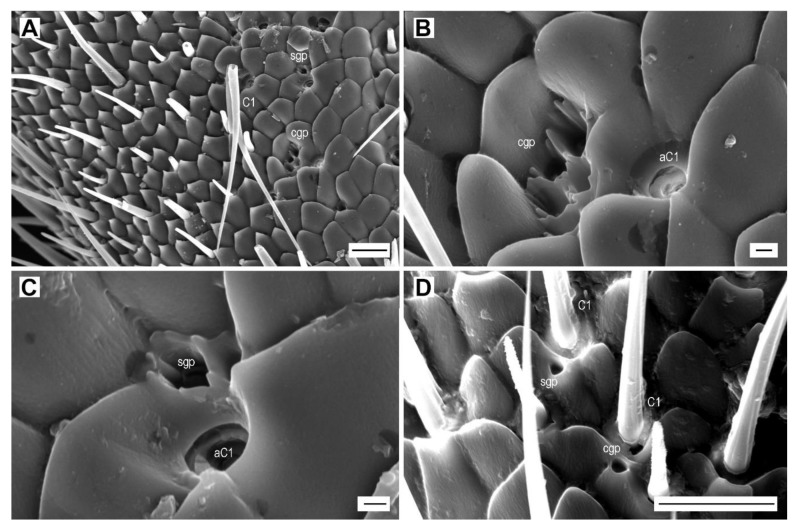
Glandular pores. (**A**) *A. pilosellus*, male, location of a single glandular pore (sgp) and a cluster of glandular pores (cgp), and sensillum chaeticum C1; (**B**) cluster of glandular pores near alveolus of sensillum C1 (aC1); (**C**) single glandular pore near alveolus of sensillum C1; (**D**) *A. medvedevi*, male, glandular pores near bases of sensilla chaetica C1. Scale bars = (**A,D**): 10 μm; (**B,C**): 1 μm.

In all examined species, the pores that could be the openings of epidermal gland ducts occur in the median portions of each antennomere, outside of the sensillar fields. They are usually located near sensilla chaetica C1. In *A. pilosellus*, they are circular, 1.8 µm in diameter, and either as a single glandular pore or as a cluster of four glandular pores (Figure 11A–C). They can be easily distinguished from the alveolus of sensillum C1 by the sharp edges with short digitations. In *A. obscurus*, these pore types are located near sensilla basiconica B7. In *A. medvedevi*, one or two semi-circular pores with a smooth edge, 1.0–1.3 µm in diameter, are located near sensilla C1 (Figure 11D).

## 4. Discussion

In this paper, we focused on the comparative morphology of antennae within the species of *Agriotes*. We studied representatives of 10 European species, of which five are among the most important pest species in Europe, with three of these also occur in North America [10,16]. Our main goal was to investigate the antennal sensilla in *Agriotes* including their typology, location, number, and possible function. We identified six main types of sensilla in *Agriotes*, including sensilla chaetica (subtypes C1 and C2), sensilla trichodea, sensilla basiconica (subtypes B1–B9), dome-shaped sensilla (subtypes D1 and D2), sensilla campaniformia, and Böhm sensilla. The majority of sensilla present on antennae of *Agriotes* spp. was observed also in other Elateridae, i.e., *Agriotes obscurus* [26,27], *Limonius aeruginosus* (Olivier, 1790) (Dendrometrinae) [28], *Melanotus villosus* (Geoffroy, 1785), and *M. cribricollis* [29,30] (both Elaterinae: Melanotini), *Tetrigus lewisi* Candèze, 1873 (Agrypninae: Ludioctenini) [31], *Elater ferrugineus* Linnaeus, 1758 (Elaterinae: Elaterini) [32], *Drilus* spp., *Malacogaster* spp., and *Selasia* spp. (Agrypninae: Drilini) [33] (Table 5). The “sensilla knee-bend shaped”, “sensilla hook-shaped”, and “sensilla screw cap” described in *Melanotus cribricollis* Candèze, 1860 by Peng et al. [30] are not sensillum types but only some sensilla trichodea or basiconica, which were probably distorted during the preparation of antennae for the SEM. The incorrect and/or inconsistent use of sensillum nomenclature, even in a relatively small taxonomic insect group, is a common phenomenon that often leads to alternative naming of single sensillum types by different authors (some examples are given in the subsequent sections) (Table 5). This makes reliable identification of sensillum types and comparisons between taxa difficult. Thus, construction of a consistent nomenclature for antennal sensillum types in insects would be of great utility [33,37].

### 4.1. Sensilla chaetica

Sensilla chaetica of subtypes C1 and C2 are present in all Coleoptera. The sensilla C1 were correctly identified as sensilla chaetica in all Elateridae so far examined in previous studies [26,27,28,29,30,31,32,33,38,39] (Table 5). As in other click-beetles [32], these sensilla are widely and evenly distributed on antennae and are more abundant in females. Sensilla C1 are tactile mechanoreceptors responding to mechanical stimuli and enabling the beetle to determine the position of the antennae with respect to its surroundings [28,36,40,41].

Within Elateridae, the sensilla chaetica C2 of *Agriotes* spp. are identical to the sensilla named as trichodea T1 in *Agriotes obscurus*, *Tetrigus lewisi,* and *Elater ferrugineus* [26,31,32], trichodea in *Limonius aeruginosus* [28], trichodea type III in *Melanotus villosus* [29], and chaetica C2 in Drilini [33]. Further, these sensilla are morphologically similar to the sensilla described on the antennal flagellum of various other beetle lineages, e.g., sensilla chaetica C2 of *Lampyris noctiluca* Linnaeus, 1767 (Lampyridae) [42], sensilla trichodea of *Monochamus* spp. [43] and *Psacothea hilaris* (Pascoe, 1857) [44], sensilla chaetica with a terminal pore of *Cerambyx cerdo* Linnaeus, 1758 [45], the uniporous sensilla chaetica of *Phoracantha recurva* Newman, 1840 [46] (all Cerambycidae), sensilla trichodea TII of *Curculio caryae* Horn, 1873 [47], sensilla trichodea TIII of *Ips pini* (Say, 1826) [48] (both Curculionidae), and sensilla chaetica ch2 of *Bembidion* (*Metallina*) *lampros* (Herbst, 1784) [49] and *Anchomenus dorsalis* (as *Platynus*) (Pontoppidan, 1763) [50] (both Carabidae). The terminal pore was observed in this sensillum subtype in previous studies [45,46,48] and it is hypothesized in *Agriotes* as well. The apical finger-like processes observed in sensilla C2 are common in the uniporous sensilla chaetica in Lepidoptera [36]. Consequently, these sensilla probably have a gustatory function as they have several characteristics in common with the contact chemoreceptors of many other insects, such as a terminal pore at the blunt tip, and because they protrude above all other antennal sensilla [36,41]. Furthermore, it appears that the gustatory function of these sensilla is more effective in females that always bear higher numbers of these sensilla than males do. Because of their position at a right angle to the surface, these sensilla allow the antennomeres to easily enter into contact with the substrates.

### 4.2. Sensilla trichodea

Sensilla trichodea of the here-examined *Agriotes* species agree with the sensilla trichodea type II (T2) of *Agriotes obscurus* [26,27] and *Elater ferrugineus* [32] (Table 5). These sensilla were shown by means of electrophysiological methods to be pheromone receptors in *Agriotes* [27]. This is in agreement with studies on other insects, including moths, in which these sensilla were assumed to have a sex pheromone-receptive function [36,51,52]. This is further supported by the fact that sensilla trichodea are always much more numerous in males than in females (Tables S2–S11). The volatile sex pheromone components were identified for females of several *Agriotes* species including *A. obscurus*, *A. lineatus*, *A. brevis*, *A. sputator*, and *A. ustulatus* [20,21,22,23]. The presence of sensilla trichodea in female insects is difficult to explain easily and needs further investigation. There is no obvious explanation for female response to female pheromones [53] but it has been demonstrated that female sensilla trichodea are sensitive to the female pheromones, although less so than those of males, and they appear to be sensitive mainly to the odors of the host plants [54,55].

### 4.3. Sensilla basiconica

Sensilla basiconica form up to 38.4% of the total sensilla in *Agriotes* males and up to 43.2% in females (Table 4). They represent the major portion of the olfactory receptor system on the antennae of *Agriotes*. The olfactory function of these sensilla is obvious from the numerous wall pores [35,41]. Sensilla basiconica in insects were found to respond to host plant odors, and each of the subtypes can be specialized for a particular set of host and non-host volatiles [56,57,58].

In *Agriotes*, we identified seven subtypes that occur in all examined species and two additional ones present only in some species (Table 3). There are two main subtypes, i.e., sensilla B1 and B2, which are by far the most numerous sensilla basiconica. The clusters of these sensilla in ventral sensillar fields suggest that they play an important role in an olfactory search for food plants during the flight. They were also identified in other Elateridae [26,27,28,29,30,31,32,33]. Multiporous sensilla B3 and B4 are similar to sensilla B1 and B2 and most probably have the same olfactory function. This is further supported by their position on the distal part of the antenna. Sensilla B5 agree in their shape, location, and the relatively small number with sensilla basiconica type V (B5) of *Agriotes obscurus* and *Limonius aeruginosus* [26,28], and also with the “sensilla styloconica” of *Melanotus villosus* [29] and *Elater ferrugineus* [32]. The latter interpretation is, however, incorrect because the authors consider the nipple as the sensory cone and the cone of the sensillum as a stylus. Therefore, the sensilla B5 of *Agriotes* do not correspond to the sensilla styloconica, which were described in Lepidoptera, and do not have the same function as proposed by Merivee et al. [29]. Sensilla styloconica in moths have a thermo-hygroreceptive or gustatory function. Their apical sensory cone (perforated or not) is articulated to the stylus by a joint membrane [36]. Although we have not observed any wall pores, we believe that sensilla B5 have an olfactory function.

Regarding sensilla B6, their location only on the apical antennomere, along with the presence of wall pores, suggest a special olfactive function of this sensillum subtype in both sexes. Sensilla B7 are usually present on antennomeres IV–XI in *Agriotes*. Within the Elateridae studied so far, they correspond to sensilla basiconica type VII of *Agriotes obscurus* [26], sensilla basiconica type 4 of *Limonius aeruginosus* [28], grooved pegs of *Melanotus villosus* [29], *M. cribricollis* [30], and *Elater ferrugineus* [32], and sensilla coeloconica of *Tetrigus lewisi* [31]. Within Drilini, they are morphologically similar to sensilla basiconica 5–9, most closely resembling subtypes 7 and 8 [33]. They are also similar to the sensilla called “fluted cones” in *Ips pini* (Say, 1826) (Curculionidae: Scolytinae) [48] or “double-walled sensilla” in *Ips typographus* (Linnaeus, 1758) (Curculionidae: Scolytinae) and *Cerambyx cerdo* (Linnaeus, 1758) (Cerambycidae) [46,59]. These characteristic multiporous double-walled sensilla respond to odors, especially to short-chain n-acids and amines [40,60]. The sensillum basiconicum B8 is a large, abnormal sensory structure that was found only on antennomere X in a single male of *A. lineatus* from Romania. As far as we know, this sensillum has not been described in any other insects except a male of *Drilus concolor* Ahrens, 1812 (Elateridae: Drilini), in which it was identified as sensillum basiconicum type 16 [33]. It was approximately three times smaller (9 µm) and located on antennomere VIII. With its latero-apical position on the pre-apical antennomere, the sensillum B8 in *A. lineatus* resembles a “large sensillum basiconicum” present on the female antenna of *Drilus mauritanicus* Lucas, 1842 [38] and a “multiporous basiconic sensillum complex” present on the female antenna of *Malacogaster nigripes* Schaufuss, 1867 (both Elateridae: Drilini) [61]. The olfactory sensilla basiconica B9 of *A. pilosellus* resemble sensilla basiconica B10 of *Malacogaster passerinii* [33], sensilla B3 of the cerambycid *Xylotrechus grayii* (White, 1855) [37], and sensilla auricillica of *Tetrigus lewisi* [31].

### 4.4. Dome-shaped sensilla

Dome-shaped sensilla D1 were identified in various beetles, including all Elateridae studied so far. However, they were misidentified or differently named in earlier Elateridae studies, which needs some explanation [62]. Merivee [26] and Merivee et al. [27] examined the antennal sensillar equipment of *A. obscurus* and they identified the dome-shaped sensilla correctly. Later, Merivee et al. [28,29] identified identical sensilla on the antennae of *Limonius aeruginosus* and *Melanotus villosus* as sensilla campaniformia. Their misidentifications were followed by Ren et al. [31] for *Tetrigus lewisi* and by Zauli et al. [32] for *Elater ferrugineus*. Faucheux and Kundrata [33] referred to dome-shaped sensilla as basiconica B14 in Agrypninae: Drilini. Misinterpretation of dome-shaped sensilla as sensilla campaniformia (or their synonymy [29]) also had implications for their hypothesized possible functions [62]. In fact, the differences between these types of sensilla are obvious. The dome-shaped sensilla are “wall-pore sensilla” [35,40] or “multiporous sensilla” [34,41] with a short sensory cone located apically on a dome (Figure 9). Recent studies showed that they possibly have olfactory and thermo/hygroreceptive functions [33,63]. Interestingly, morphologically similar sensilla were identified to detect atmospheric carbon dioxide in adults of various insect groups. These structures are usually composed of clusters of wall-pore sensilla that may form distinct sensory organs [64]. Indeed, dome-shaped sensilla usually occur in groups in *Agriotes*, as well as in, e.g., *Selasia* spp. (Elateridae: Drilini) and Curculionidae [33,47]. On the other hand, sensilla campaniformia are “no-pore sensilla” [35,40] or “aporous sensilla” [34,41] with a ring of raised cuticle (instead of a cuticular dome), and a small central cuticular cap without any pores (instead of a multiporous sensory cone) [62]. Moreover, sensilla campaniformia are usually situated near the joints on antennae and function as proprioceptors [65], which is not the case for dome-shaped sensilla in Elateridae.

Dome-shaped sensilla D2 are present only on the apical antennomeres of two *Agriotes* species. They resemble the sensilla basiconica B13 of *Drilus concolor* [33], basiconica type I of Chrysomelidae [66], and basiconica type II of Coccinellidae [67]. Following previous studies [33], we hypothesize a hygro- and/or thermoreceptive function for this sensillum subtype. 

### 4.5. Sensilla campaniformia

Sensilla campaniformia are mechanoreceptors of proprioceptive function that respond to stress and strain in the exoskeleton of insects and are mostly concentrated near the antennal joints [65,68]. In *Agriotes*, there is a single campaniform sensillum located at the distal edge of the pedicel near the joint with antennomere III in all examined specimens (Figure 10A,B; Tables S2–S11), similar to various other insects, like Lepidoptera [36], Diptera [69], or Psocoptera [70]. We have not found sensilla campaniformia with an incomplete cuticular ring as described for the flagellomeres of Drilini [33]. The sensilla designated as “campaniformia” by Merivee et al. [28,29], Peng et al. [30], Ren et al. [31], and Zauli et al. [32] in other Elateridae are in fact dome-shaped sensilla (see comments above). Therefore, the only other click-beetle species in which true sensilla campaniformia were observed has been *Drilus flavescens* (Geoffroy, 1785) [33]. The fact that sensilla campaniformia were not reported for many click-beetle species during the SEM studies can be explained by the inconspicuous morphology of these sensilla.

### 4.6. Böhm sensilla

These sensilla are typically present on the scape and pedicel in various insects, including Elateridae, and function as proprioceptors that monitor antennal movements and position [33,36,68]. In all examined *Agriotes* specimens, they were at the same locations and in similar numbers, which suggests their function is identical in all species. Although these sensilla are usually called Böhm sensilla or bristles [28,29,33], it should be noted that they were previously identified as sensilla chaetica C2 in *Agriotes obscurus* [26] (Table 5).

### 4.7. Glandular pores

Glandular pores were usually located near sensilla chaetica C1. According to Dyer and Seabrook [43], the association of glands with sensilla chaetica could prevent their obstruction. More likely, the liquid ejected by the pores could have a lubricating function [32]. We did not find the perforated plates that were reported for Drilini [33].

## 5. Conclusions

*Agriotes*, as an important agriculture pest, serves as a good model taxon for pest control and management [1,2,3,4,6]. Numerous studies focused on pheromone composition in several *Agriotes* species [11,13,14,15,16,17,18,19,20,21,22,23,24,25] in order to develop effective species-specific pheromone traps. However, our knowledge of the sensillar equipment, including the typology and functions of different sensilla, has been highly underexplored. What is more, the comparative study of different populations and species of this genus might help us to understand the inter- and intraspecific variability in the sensory systems within *Agriotes*. Therefore, the present study should provide a strong preliminary framework for more thorough research on the antennal morphology of this crop pest. 

Additionally, this study can provide important antennal morphological characters for future comprehensive systematic and phylogenetic studies that would attempt to solve the classification, evolution, and phylogenetic relationships among *Agriotes* species. Gurjeva [71] divided the Palaearctic *Agriotes* into several morphologically defined groups but some of them are intermediate between other groups, and their limits need rigorous testing. Increased knowledge of the antennal morphology of different *Agriotes* species might be the first step toward understanding the natural classification of the group. Although preliminary in number of examined species and populations, our study provides some interesting information regarding the interspecific variability in sensillar equipment in *Agriotes* (Table 3). Not taking into account the apparently abnormal sensillum basiconicum S8 in one of the males in *A. lineatus*, only *A. pilosellus* has a unique subtype of sensillum basiconicum, and *A. acuminatus* and *A. medvedevi* share the unique subtype of the dome-shaped sensillum. The slight variation of presence and absence of sensilla basiconica B3, B4, B5, and B7, and the dome-shaped sensilla D1 on certain antennomeres in some species (in most cases only in a specific sex) needs further examination. The abovementioned sensilla subtypes are present on *Agriotes* antennae only in small numbers, so it might represent only intraspecific variability rather than a species-specific character. More specimens of both sexes of a single population, as well as more geographically distant populations of each examined species, are crucial for further understanding this variability. The relative proportions of different types of sensilla in *Agriotes* also vary among species (Table 4) and might be a promising information source for future systematic and phylogenetic studies. For example, while the intraspecific variability of the proportion of sensilla chaetica from the total number of sensilla per antenna is relatively low (always less than 3%), the interspecific variability might reach up to approximately 37% (between males of *A. acuminatus* and *A. modestus*). Additionally, *A. acuminatus* is the only examined species with more sensilla basiconica than sensilla chaetica. It should be noted that *A. acuminatus* differs from the remaining examined species by its small body size. Therefore, it would be interesting to examine the sensillar equipment for some other small-sized *Agriotes* species, especially from the same group of species sensu Gurjeva [71].

Lastly, given the inconsistent use of sensillum nomenclature and difficulties associated with reliable identification of the sensillum types even within a single family (Table 5), we provided a comparison of the sensillar equipment of *Agriotes* with other Elateridae [28,29,30,31,32,33] in an attempt to consolidate the sensillum nomenclature for future studies on this family. This would also aid researchers in assigning the correct functions to identified sensilla [62].

## Figures and Tables

**Table 1:** List of *Agriotes* material used for the SEM study. M, male; F, female.

Code	Species	Sex	Geographic Origin
Ag01	*A. acuminatus*	M	Hungary, Vas county, Sárvár
Ag02	*A. acuminatus*	M	Hungary, Szabolcs-Szatmár county, Tákos
Ag03	*A. acuminatus*	M	Hungary, Somogy county, Kaposvár
Ag04	*A. acuminatus*	F	Greece, Ioannina regional unit, Metsovo
Ag05	*A. acuminatus*	F	Greece, Ioannina regional unit, Metsovo
Ag06	*A. acuminatus*	F	Hungary, Tolna county, Bátaapáti
Ag07	*A. lineatus*	M	Romania, Sălaj county, Huta
Ag08	*A. lineatus*	M	Hungary, Baranya county, Sumony
Ag09	*A. lineatus*	M	Hungary, Hajdú-Bihar county, Nagyhegyes
Ag10	*A. lineatus*	F	Hungary, Budapest
Ag11	*A. medvedevi*	M	Hungary, Bács-Kiskun county, Dunatetétlen
Ag12	*A. medvedevi*	M	Hungary, Bács-Kiskun county, Dunatetétlen
Ag13	*A. medvedevi*	M	Hungary, Bács-Kiskun county, Dunatetétlen
Ag14	*A. medvedevi*	M	Hungary, Bács-Kiskun county, Dunatetétlen
Ag15	*A. medvedevi*	F	Hungary, Bács-Kiskun county, Dunatetétlen
Ag16	*A. medvedevi*	F	Hungary, Bács-Kiskun county, Dunatetétlen
Ag17	*A. medvedevi*	F	Hungary, Bács-Kiskun county, Dunatetétlen
Ag18	*A. modestus*	M	Hungary, Pest county, Tatárszentgyörgy
Ag19	*A. modestus*	M	Hungary, Pest county, Tatárszentgyörgy
Ag20	*A. modestus*	F	Hungary, Pest county, Tatárszentgyörgy
Ag21	*A. obscurus*	M	Czech Republic, Moravia, Olomouc
Ag22	*A. obscurus*	M	Czech Republic, Moravia, Olomouc
Ag23	*A. obscurus*	M	Czech Republic, Moravia, Olomouc
Ag24	*A. obscurus*	M	Hungary, Pest county, Tahitótfalu
Ag25	*A. obscurus*	F	Romania, Sălaj county, Huta
Ag26	*A. obscurus*	F	Hungary, Borsod-Abaúj-Zemplén county, Szögliget
Ag41	*A. paludum*	M	Greece, Corinthia regional unit, Kato Sinikia Trikala
Ag42	*A. paludum*	F	Greece, Grevena regional unit, Kipuro
Ag27	*A. pilosellus*	M	Romania, Sălaj county, Aghireș
Ag28	*A. pilosellus*	M	Hungary, Pest county, Kemence
Ag29	*A. pilosellus*	F	Hungary, Győr-Moson-Sopron county, Sopron
Ag30	*A. rufipalpis*	M	Hungary, Heves county, Hort
Ag32	*A. rufipalpis*	M	Albania, Shkodër county, Omarë
Ag31	*A. rufipalpis*	F	Albania, Shkodër county, Omarë
Ag33	*A. sputator*	M	Hungary, Bács-Kiskun county, Dunatetétlen
Ag34	*A. sputator*	M	Hungary, Bács-Kiskun county, Dunatetétlen
Ag35	*A. sputator*	F	Hungary, Bács-Kiskun county, Dunatetétlen
Ag36	*A. sputator*	F	Hungary, Bács-Kiskun county, Dunatetétlen
Ag37	*A. ustulatus*	M	Czech Republic, Moravia, Olomouc
Ag38	*A. ustulatus*	M	Hungary, Pest county, Vác
Ag39	*A. ustulatus*	F	Albania, Shkodër county, Lisi i Locit
Ag40	*A. ustulatus*	F	Hungary, Heves county, Kerecsend

**Table 2:** Morphological characteristics of different sensillum types and subtypes in *Agriotes* species. ?, pores hypothesized but not clearly visible in the studied material.

Sensillum Type	Length (µm)	Basal Width (µm)	Pores
chaeticum C1	75.6 ± 4.8	3.3 ± 0.3	no pore
chaeticum C2	87.8 ± 2.4	4.2 ± 0.4	terminal pore?
trichodeum	24.6 ± 3.9	2.3 ± 0.2	wall pores?
basiconicum B1	10.9 ± 1.7	2.1 ± 0.3	wall pores
basiconicum B2	11.5 ± 2.3	2.0 ± 0.1	wall pores
basiconicum B3	10.1 ± 0.9	1.8 ± 0.2	wall pores
basiconicum B4	6.2 ± 0.8	1.6 ± 0.2	wall pores
basiconicum B5	5.8 ± 0.7	1.8 ± 0.3	wall pores?
basiconicum B6	9.7 ± 0.6	1.9 ± 0.1	wall pores
basiconicum B7	5.1 ± 0.3	1.8 ± 0.4	wall pores
basiconicum B8	27.3 ± 0.1	12.5 ± 0.1	wall pores?
basiconicum B9	9.9 ± 0.3	2.2 ± 0.2	wall pores?
dome-shaped D1	5.1 ± 0.4	5.8 ± 0.4	wall pores
dome-shaped D2	3.8 ± 0.3	3.6 ± 0.5	wall pores
Böhm sensillum	8.7 ± 2.3	1.8 ± 0.3	no pore

**Table 3:** Presence (+) or absence (-) of sensillum types and subtypes on different antennomeres of the examined *Agriotes* species. SC1 and SC2, sensilla chaetica subtypes 1 and 2, respectively; ST, sensilla trichodea; SB1–9, sensilla basiconica subtypes 1–9; SD1–2, dome-shaped sensilla subtypes 1–2; SCa, sensilla campaniformia; BS, Böhm sensilla.

Sensilla	Antennomere										
	I	II	III	IV	V	VI	VII	VIII	IX	X	XI
SC1	+	+	+	+	+	+	+	+	+	+	+
SC2	+	+	+	+	+	+	+	+	+	+	+
ST	-	-	-	+	+	+	+	+	+	+	+
SB1	-	-	-	+	+	+	+	+	+	+	+
SB2	-	-	-	+	+	+	+	+	+	+	+
SB3	-	-	-	-	-	-	-	-	-	+^1^	+
SB4	-	-	-	-	-	-	+^2^	+	+	+	+
SB5	-	-	-	-	-	-	-	-	-	+^3^	+
SB6	-	-	-	-	-	-	-	-	-	-	+
SB7	-	-	-	+	+^4^	+^5^	+^6^	+	+	+	+
SB8	-	-	-	-	-	-	-	+^7^	-	-	-
SB9	-	-	-	-	-	-	-	-	+^8^	+^8^	+^8^
SD1	-	-	-	+	+	+^9^	+	+^10^	+	+	+
SD2	-	-	-	-	-	-	-	-	-	-	+^11^
Sca	-	+	-	-	-	-	-	-	-	-	-
BS	+	+	-	-	-	-	-	-	-	-	-

^1^ missing in both sexes of *A. lineatus* and males of *A. medvedevi*, *A. paludum*, and *A. pilosellus*; ^2^ missing in males of *A. acuminatus*, *A. medvedevi*, *A. modestus*, *A. rufipalpis*, *A. sputator*, and *A. ustulatus*; ^3^ missing in both sexes of *A. acuminatus* and *A. paludum*, and females of *A. medvedevi* and *A. sputator*; ^4^ missing in females of *A. acuminatus*; ^5^ missing in females of *A. sputator*; ^6^ missing in males of *A. medvedevi*; ^7^ present only in a single male of *A. lineatus* from Romania; ^8^ present only in *A. pilosellus*; ^9^ missing in males of *A. ustulatus*; ^10^ missing in males of *A. medvedevi*; ^11^ present only in *A. acuminatus* and *A. medvedevi*.

**Table 4:** Percentages of different sensillum types within the individual specimens of all examined *Agriotes* species. M, male; F, female; SC1 and SC2, sensilla chaetica subtypes 1 and 2, respectively; ST, sensilla trichodea; SB1–9, sensilla basiconica subtypes 1–9; SD1–2, dome-shaped sensilla subtypes 1–2; SCa, sensilla campaniformia; BS, Böhm sensilla.

Species	Code	Sex	SC1	SC2	ST	SB1–9	SD1–2	SCa	BS
*A. acuminatus*	Ag01	M	24.0%	4.3%	24.6%	38.4%	1.3%	0.1%	7.3%
	Ag02	M	25.8%	4.3%	23.9%	37.2%	1.3%	0.1%	7.4%
	Ag03	M	24.7%	4.4%	26.5%	35.8%	1.2%	0.1%	7.3%
	Ag04	F	39.6%	4.4%	6.4%	41.9%	1.3%	0.1%	6.3%
	Ag05	F	38.2%	4.4%	6.6%	43.2%	1.3%	0.1%	6.2%
	Ag06	F	39.3%	4.5%	5.9%	42.6%	1.3%	0.1%	6.3%
*A. lineatus*	Ag07	M	46.3%	2.8%	20.7%	25.1%	0.9%	<0.1%	4.3%
	Ag08	M	45.4%	3.2%	19.3%	26.6%	1.0%	<0.1%	4.5%
	Ag09	M	45.9%	3.0%	20.4%	25.4%	0.9%	<0.1%	4.4%
	Ag10	F	54.6%	3.4%	3.4%	32.9%	1.1%	<0.1%	4.5%
*A. medvedevi*	Ag11	M	51.6%	2.3%	17.5%	23.6%	0.8%	<0.1%	4.3%
	Ag12	M	52.0%	2.2%	18.1%	22.5%	0.9%	<0.1%	4.3%
	Ag13	M	51.4%	2.3%	17.7%	23.3%	0.9%	<0.1%	4.4%
	Ag14	M	51.8%	2.3%	18.1%	22.4%	1.0%	<0.1%	4.4%
	Ag15	F	59.2%	2.7%	2.9%	30.2%	0.9%	<0.1%	4.1%
	Ag16	F	58.7%	2.5%	3.2%	30.5%	0.9%	<0.1%	4.2%
	Ag17	F	60.2%	2.4%	3.0%	29.2%	0.9%	<0.1%	4.2%
*A. modestus*	Ag18	M	60.6%	2.0%	14.5%	18.6%	0.7%	<0.1%	3.5%
	Ag19	M	59.8%	2.1%	13.9%	19.8%	0.8%	<0.1%	3.6%
	Ag20	F	65.3%	2.3%	4.7%	23.3%	0.9%	<0.1%	3.4%
*A. obscurus*	Ag21	M	49.8%	2.4%	20.5%	22.6%	1.0%	<0.1%	3.7%
	Ag22	M	50.9%	2.3%	20.4%	21.7%	1.0%	<0.1%	3.7%
	Ag23	M	51.0%	2.2%	20.4%	21.4%	1.1%	<0.1%	3.9%
	Ag24	M	47.3%	2.3%	19.9%	25.7%	1.0%	<0.1%	3.8%
	Ag25	F	60.9%	2.7%	4.6%	27.0%	1.0%	<0.1%	3.7%
	Ag26	F	59.4%	2.6%	4.8%	28.2%	1.1%	<0.1%	3.9%
*A. paludum*	Ag41	M	45.7%	3.1%	20.8%	25.2%	1.0%	<0.1%	4.4%
	Ag42	F	54.9%	3.3%	4.4%	32.3%	0.8%	<0.1%	4.2%
*A. pilosellus*	Ag27	M	41.0%	2.1%	19.3 %	32.9%	0.8%	<0.1%	4.0%
	Ag28	M	39.7%	2.1%	19.3%	34.2%	0.7%	<0.1%	4.0%
	Ag29	F	49.6%	2.5%	3.8%	39.4%	0.7%	<0.1%	4.0%
*A. rufipalpis*	Ag30	M	46.4%	2.8%	16.7%	28.4%	1.0%	<0.1%	4.7%
	Ag32	M	48.7%	2.6%	15.3%	27.7%	1.0%	<0.1%	4.7%
	Ag31	F	51.2%	3.0%	3.9%	36.5%	0.8%	<0.1%	4.5%
*A. sputator*	Ag33	M	46.3%	2.6%	18.8%	27.1%	0.8%	<0.1%	4.3%
	Ag34	M	45.9%	2.5%	19.2%	27.3%	0.8%	<0.1%	4.3%
	Ag35	F	55.7%	3.1%	3.5%	32.5%	0.8%	<0.1%	4.4%
	Ag36	F	51.8%	2.9%	3.2%	37.0%	0.8%	<0.1%	4.3%
*A. ustulatus*	Ag37	M	37.0%	3.4%	24.9%	28.4%	1.0%	<0.1%	5.2%
	Ag38	M	38.2%	3.5%	26.1%	25.9%	1.1%	<0.1%	5.2%
	Ag39	F	46.0%	4.2%	6.9%	36.3%	1.0%	<0.1%	5.4%
	Ag40	F	45.5%	4.2%	7.3%	36.7%	1.0%	<0.1%	5.3%

**Table 5:** Sensillum types and subtypes in Elateridae by different authors. campan., campaniformia.

This Study *Agriotes*	[26,27] *Agriotes*	[28,29] Limonius*, Melanotus**	[31] *Tetrigus*	[32] *Elater*	[33] Drilini
chaetica 1	chaetica 1	chaetica	chaetica 1,2,3	chaetica	chaetica 1
chaetica 2	trichodea 1	trichodea*, trichodea III**	trichodea 1	trichodea 1	chaetica 2
trichodea	trichodea 2	–––	–––	trichodea 2	–––
basiconica 1	basiconica 1	basiconica 1*, basiconica 2**	basiconica 1	basiconica 1	basiconica 1
basiconica 2	basiconica 2	basiconica 2*, basiconica 1**	basiconica 2	basiconica 2	basiconica 2
basiconica 3	basiconica 3	basiconica 3*	–––	–––	–––
basiconica 4	basiconica 4	–––	–––	–––	–––
basiconica 5	basiconica 5	basiconica 5*, styloconica**	–––	styloconica	–––
basiconica 6	basiconica 6	–––	–––	–––	–––
basiconica 7	basiconica 7	basiconica 4*	coeloconica	grooved pegs	basiconica 7,8
basiconica 8	–––	–––	–––	–––	basiconica 16
basiconica 9	–––	–––	–––	–––	basiconica 10
dome-shaped 1	dome-shaped	campan. dome-shaped**	campan.	campan.	basiconica 14
dome-shaped 2	–––	–––	–––	–––	basiconica 13
campaniformia	–––	–––	–––	–––	campan.
Böhm sensilla	chaetica 2	Böhm sensilla or bristles**	Böhm’s bristles	Böhm sensilla	Böhm sensilla

## Supplementary Materials

The following are available online at https://www.mdpi.com/2075-4450/11/2/137/s1, Table S1: The relative ratio of antennomere lengths (I–XI) and the total antennal lengths (in mm) for the examined *Agriotes* species, Table S2: Distribution and numbers of different sensilla on the antennomeres I–XI in *Agriotes acuminatus*, Table S3: Distribution and numbers of different sensilla on the antennomeres I–XI in *Agriotes lineatus*, Table S4: Distribution and numbers of different sensilla on the antennomeres I–XI in *Agriotes medvedevi*, Table S5: Distribution and numbers of different sensilla on the antennomeres I–XI in *Agriotes modestus*, Table S6: Distribution and numbers of different sensilla on the antennomeres I–XI in *Agriotes obscurus*, Table S7: Distribution and numbers of different sensilla on the antennomeres I–XI in *Agriotes paludum*, Table S8: Distribution and numbers of different sensilla on the antennomeres I–XI in *Agriotes pilosellus*, Table S9: Distribution and numbers of different sensilla on the antennomeres I–XI in *Agriotes rufipalpis*, Table S10: Distribution and numbers of different sensilla on the antennomeres I–XI in *Agriotes sputator*, Table S11: Distribution and numbers of different sensilla on the antennomeres I–XI in *Agriotes ustulatus*.
