# Supplementary material for: Comparative Antennal Morphology of Agriotes (Coleoptera: Elateridae), with Special Reference to the Typology and Possible Functions of Sensilla
Source: Insects. 2020 Feb 21;11(2):137. doi: 10.3390/insects11020137 (PMC7074560; doi:10.3390/insects11020137)
Supplement: Supplementary file 1 [file insects-11-00137-s001.pdf]

Comparative antennal morphology of *Agriotes* (Coleoptera: Elateridae), with special reference to the sensilla

Michel J. Faucheux, Tamás Németh and Robin Kundrata

List of Supplementary Files

Table S1. The relative ratio of antennomere lengths (I–XI) and the total antennal lengths (in mm) for the examined *Agriotes* species.
Table S2. Distribution and numbers of different sensilla on the antennomeres I–XI in *Agriotes acuminatus*.
Table S3. Distribution and numbers of different sensilla on the antennomeres I–XI in *Agriotes lineatus*.
Table S4. Distribution and numbers of different sensilla on the antennomeres I–XI in *Agriotes medvedevi*.
Table S5. Distribution and numbers of different sensilla on the antennomeres I–XI in *Agriotes modestus*.
Table S6. Distribution and numbers of different sensilla on the antennomeres I–XI in *Agriotes obscurus*.
Table S7. Distribution and numbers of different sensilla on the antennomeres I–XI in *Agriotes paludum*.
Table S8. Distribution and numbers of different sensilla on the antennomeres I–XI in *Agriotes pilosellus*.
Table S9. Distribution and numbers of different sensilla on the antennomeres I–XI in *Agriotes rufipalpis*.
Table S10. Distribution and numbers of different sensilla on the antennomeres I–XI in *Agriotes sputator*.
Table S11. Distribution and numbers of different sensilla on the antennomeres I–XI in *Agriotes ustulatus*.

Table S1. The relative ratio of antennomere lengths (I–XI) and the total antennal lengths (in mm) for the examined *Agriotes* species.

Species	Sex	Relative ratio of antennomere lengths	Total length
A. acuminatus	M	2.8 : 1.7 : 1.0 : 1.6 : 1.7 : 1.7 : 1.7 : 1.7 : 1.7 : 1.8 : 2.7	1.9
	F	3.0 : 1.9 : 1.0 : 1.7 : 1.7 : 1.7 : 1.8 : 1.7 : 1.8 : 1.7 : 2.6	1.9
A. lineatus	M	2.0 : 1.4 : 1.0 : 1.2 : 1.1 : 1.3 : 1.2 : 1.2 : 1.2 : 1.3 : 1.6	3.3
	F	2.1 : 1.6 : 1.0 : 1.0 : 1.0 : 1.1 : 1.2 : 1.2 : 1.4 : 1.5 : 2.0	2.8
A. medvedevi	M	2.6 : 1.6 : 1.0 : 1.3 : 1.3 : 1.6 : 1.6 : 1.7 : 1.7 : 1.5 : 2.3	2.7
	F	2.2 : 1.6 : 1.0 : 1.4 : 1.4 : 1.4 : 1.4 : 1.3 : 1.5 : 1.4 : 2.2	2.4
A. modestus	M	2.0 : 1.2 : 1.0 : 1.3 : 1.2 : 1.3 : 1.3 : 1.2 : 1.3 : 1.3 : 1.6	4.4
	F	1.8 : 1.1 : 1.0 : 1.2 : 1.2 : 1.1 : 1.2 : 1.3 : 1.3 : 1.3 : 1.4	4.0
A. obscurus	M	2.5 : 1.5 : 1.0 : 1.2 : 1.2 : 1.4 : 1.4 : 1.5 : 1.5 : 1.7 : 1.9	3.5
	F	2.8 : 1.4 : 1.0 : 1.3 : 1.3 : 1.4 : 1.4 : 1.3 : 1.4 : 1.7 : 2.1	3.0
A. paludum	M	2.2 : 1.5 : 1.0 : 1.1 : 1.2 : 1.2 : 1.2 : 1.3 : 1.4 : 1.6 : 1.8	3.2
	F	2.2 : 1.6 : 1.0 : 1.3 : 1.3 : 1.4 : 1.4 : 1.4 : 1.5 : 1.6 : 2.0	3.0
A. pilosellus	M	1.8 : 1.2 : 1.0 : 1.1 : 1.1 : 1.3 : 1.3 : 1.3 : 1.4 : 1.4 : 1.9	4.5
	F	1.8 : 1.2 : 1.0 : 1.4 : 1.3 : 1.4 : 1.3 : 1.5 : 1.5 : 1.4 : 2.0	4.5
A. rufipalpis	M	1.9 : 1.6 : 1.0 : 1.3 : 1.3 : 1.1 : 1.2 : 1.3 : 1.3 : 1.2 : 1.8	2.8
	F	1.7 : 1.4 : 1.0 : 1.5 : 1.2 : 1.3 : 1.2 : 1.4 : 1.4 : 1.3 : 1.6	2.9
A. sputator	M	2.9 : 1.8 : 1.0 : 1.4 : 1.6 : 1.5 : 1.4 : 1.6 : 1.8 : 1.7 : 2.7	2.2
	F	2.3 : 1.3 : 1.0 : 1.2 : 1.2 : 1.3 : 1.2 : 1.3 : 1.4 : 1.5 : 1.9	2.2
A. ustulatus	M	2.5 : 1.7 : 1.0 : 1.2 : 1.1 : 1.2 : 1.3 : 1.3 : 1.3 : 1.4 : 2.0	2.9
	F	2.3 : 1.6 : 1.0 : 1.2 : 1.1 : 1.2 : 1.2 : 1.2 : 1.3 : 1.3 : 2.0	2.8

Table S2. Distribution and numbers of different sensilla on the antennomeres I–XI in *Agriotes acuminatus* (Ag01, Ag04). M, male; F, female; SC1 and SC2, sensilla chaetica subtypes 1 and 2, respectively; ST, sensilla trichodea; SB1–9, sensilla basiconica subtypes 1–9; SD1–2, dome-shaped sensilla subtypes 1–2; SCa, sensilla campaniformia; BS, Böhm sensilla.

**Sensilla**	**Sex**	**Antennomere**											
		**I**	**II**	**III**	**IV**	**V**	**VI**	**VII**	**VIII**	**IX**	**X**	**XI**	**Total**
SC1	M	78	37	17	24	30	35	32	26	33	29	42	383
	F	89	46	29	63	69	76	56	62	72	67	91	720
SC2	M	4	5	6	6	6	6	6	7	6	7	10	69
	F	4	5	7	7	7	7	8	6	7	8	13	79
ST	M	-	-	-	15	26	32	40	44	68	77	92	394
	F	-	-	-	10	12	13	15	20	18	15	13	116
SB1	M	-	-	-	40	52	36	47	45	50	54	47	371
	F	-	-	-	50	59	53	59	67	44	83	96	511
SB2	M	-	-	-	14	24	18	21	25	25	28	23	178
	F	-	-	-	12	19	16	24	27	22	35	28	183
SB3	M	-	-	-	-	-	-	-	-	-	1	6	7
	F	-	-	-	-	-	-	-	-	-	1	9	10
SB4	M	-	-	-	-	-	-	-	3	5	4	7	19
	F	-	-	-	-	-	-	2	4	6	5	8	25
SB5	M	-	-	-	-	-	-	-	-	-	-	5	5
	F	-	-	-	-	-	-	-	-	-	-	3	3
SB6	M	-	-	-	-	-	-	-	-	-	-	3	3
	F	-	-	-	-	-	-	-	-	-	-	5	5
SB7	M	-	-	-	3	5	5	4	3	3	2	6	31
	F	-	-	-	6	-	3	-	3	2	3	8	25
SB8	M	-	-	-	-	-	-	-	-	-	-	-	-
	F	-	-	-	-	-	-	-	-	-	-	-	-
SB9	M	-	-	-	-	-	-	-	-	-	-	-	-
	F	-	-	-	-	-	-	-	-	-	-	-	-
SD1	M	-	-	-	1	2	1	3	2	4	2	5	20
	F	-	-	-	2	2	1	3	3	4	3	5	23
SD2	M	-	-	-	-	-	-	-	-	-	-	2	2
	F	-	-	-	-	-	-	-	-	-	-	2	2
SCa	M	-	1	-	-	-	-	-	-	-	-	-	1
	F	-	1	-	-	-	-	-	-	-	-	-	1
BS	M	74	42	-	-	-	-	-	-	-	-	-	116
	F	73	42	-	-	-	-	-	-	-	-	-	115

Table S3. Distribution and numbers of different sensilla on the antennomeres I–XI in *Agriotes lineatus* (Ag07, Ag10). M, male; F, female; SC1 and SC2, sensilla chaetica subtypes 1 and 2, respectively; ST, sensilla trichodea; SB1–9, sensilla basiconica subtypes 1–9; SD1–2, dome-shaped sensilla subtypes 1–2; SCa, sensilla campaniformia; BS, Böhm sensilla.

**Sensilla**	**Sex**	**Antennomere**											
		**I**	**II**	**III**	**IV**	**V**	**VI**	**VII**	**VIII**	**IX**	**X**	**XI**	**Total**
SC1	M	99	64	61	95	106	110	123	137	140	148	177	1260
	F	117	68	74	100	118	129	125	142	153	161	198	1385
SC2	M	4	3	5	6	6	7	8	9	8	8	11	75
	F	4	5	6	8	7	7	8	9	9	9	14	86
ST	M	-	-	-	12	35	44	58	62	75	89	188	563
	F	-	-	-	7	9	10	9	11	14	15	12	87
SB1	M	-	-	-	29	43	53	52	31	54	67	72	401
	F	-	-	-	30	47	55	63	61	74	82	77	489
SB2	M	-	-	-	14	24	21	29	47	35	29	27	226
	F	-	-	-	18	32	38	32	30	35	37	48	270
SB3	M	-	-	-	-	-	-	-	-	-	-	7	7
	F	-	-	-	-	-	-	-	-	-	-	9	9
SB4	M	-	-	-	-	-	-	1	2	4	3	6	16
	F	-	-	-	-	-	-	3	3	6	5	7	24
SB5	M	-	-	-	-	-	-	-	-	-	2	6	8
	F	-	-	-	-	-	-	-	-	-	1	4	5
SB6	M	-	-	-	-	-	-	-	-	-	-	2	2
	F	-	-	-	-	-	-	-	-	-	-	5	5
SB7	M	-	-	-	1	2	2	3	2	2	2	8	22
	F	-	-	-	2	2	4	3	3	2	3	13	32
SB8	M	-	-	-	-	-	-	-	1	-	-	-	1
	F	-	-	-	-	-	-	-	-	-	-	-	-
SB9	M	-	-	-	-	-	-	-	-	-	-	-	-
	F	-	-	-	-	-	-	-	-	-	-	-	-
SD1	M	-	-	-	1	3	2	1	2	2	3	11	25
	F	-	-	-	2	3	2	3	3	3	4	9	29
SD2	M	-	-	-	-	-	-	-	-	-	-	-	-
	F	-	-	-	-	-	-	-	-	-	-	-	-
SCa	M	-	1	-	-	-	-	-	-	-	-	-	1
	F	-	1	-	-	-	-	-	-	-	-	-	1
BS	M	79	38	-	-	-	-	-	-	-	-	-	117
	F	77	38	-	-	-	-	-	-	-	-	-	115

Table S4. Distribution and numbers of different sensilla on the antennomeres I–XI in *Agriotes medvedevi* (Ag11, Ag15). M, male; F, female; SC1 and SC2, sensilla chaetica subtypes 1 and 2, respectively; ST, sensilla trichodea; SB1–9, sensilla basiconica subtypes 1–9; SD1–2, dome-shaped sensilla subtypes 1–2; SCa, sensilla campaniformia; BS, Böhm sensilla.

**Sensilla**	**Sex**	**Antennomere**											
		**I**	**II**	**III**	**IV**	**V**	**VI**	**VII**	**VIII**	**IX**	**X**	**XI**	**Total**
SC1	M	108	75	66	113	111	123	149	151	156	147	193	1392
	F	122	84	79	124	135	152	172	165	180	189	235	1637
SC2	M	3	4	5	5	6	5	6	6	5	7	9	61
	F	4	4	6	7	7	7	7	7	8	8	11	76
ST	M	-	-	-	10	23	41	56	62	65	78	137	472
	F	-	-	-	6	7	9	12	15	9	11	10	79
SB1	M	-	-	-	23	45	51	42	40	55	49	57	362
	F	-	-	-	38	65	68	54	56	75	68	73	497
SB2	M	-	-	-	17	23	19	26	15	37	39	40	216
	F	-	-	-	12	20	22	30	17	42	71	49	263
SB3	M	-	-	-	-	-	-	-	-	-	-	5	5
	F	-	-	-	-	-	-	-	-	-	2	11	13
SB4	M	-	-	-	-	-	-	-	3	4	3	7	17
	F	-	-	-	-	-	-	1	3	7	4	8	23
SB5	M	-	-	-	-	-	-	-	-	-	1	6	7
	F	-	-	-	-	-	-	-	-	-	-	5	5
SB6	M	-	-	-	-	-	-	-	-	-	-	4	4
	F	-	-	-	-	-	-	-	-	-	-	6	6
SB7	M	-	-	-	2	2	3	-	4	2	4	9	26
	F	-	-	-	3	2	2	3	2	2	2	12	28
SB8	M	-	-	-	-	-	-	-	-	-	-	-	-
	F	-	-	-	-	-	-	-	-	-	-	-	-
SB9	M	-	-	-	-	-	-	-	-	-	-	-	-
	F	-	-	-	-	-	-	-	-	-	-	-	-
SD1	M	-	-	-	2	3	1	2	-	2	3	7	20
	F	-	-	-	2	3	2	2	1	2	3	8	23
SD2	M	-	-	-	-	-	-	-	-	-	-	2	2
	F	-	-	-	-	-	-	-	-	-	-	2	2
SCa	M	-	1	-	-	-	-	-	-	-	-	-	1
	F	-	1	-	-	-	-	-	-	-	-	-	1
BS	M	79	37	-	-	-	-	-	-	-	-	-	116
	F	75	38	-	-	-	-	-	-	-	-	-	113

Table S5. Distribution and numbers of different sensilla on the antennomeres I–XI in *Agriotes modestus* (Ag18, Ag20). M, male; F, female; SC1 and SC2, sensilla chaetica subtypes 1 and 2, respectively; ST, sensilla trichodea; SB1–9, sensilla basiconica subtypes 1–9; SD1–2, dome-shaped sensilla subtypes 1–2; SCa, sensilla campaniformia; BS, Böhm sensilla.

**Sensilla**	**Sex**	**Antennomere**											
		**I**	**II**	**III**	**IV**	**V**	**VI**	**VII**	**VIII**	**IX**	**X**	**XI**	**Total**
SC1	M	105	52	108	164	177	184	208	215	239	242	310	2004
	F	114	73	118	170	187	198	205	220	264	291	332	2172
SC2	M	5	4	6	4	5	7	7	6	7	7	9	67
	F	6	6	4	6	6	7	7	8	8	8	11	77
ST	M	-	-	-	15	18	57	68	70	72	79	102	481
	F	-	-	-	13	15	16	19	21	24	22	25	155
SB1	M	-	-	-	10	25	39	42	50	58	47	62	333
	F	-	-	-	18	38	41	59	72	84	70	75	457
SB2	M	-	-	-	4	16	22	27	31	36	38	41	215
	F	-	-	-	9	20	21	32	38	35	41	49	245
SB3	M	-	-	-	-	-	-	-	-	-	1	7	8
	F	-	-	-	-	-	-	-	-	-	3	8	11
SB4	M	-	-	-	-	-	-	-	3	6	4	7	20
	F	-	-	-	-	-	-	1	2	6	4	9	22
SB5	M	-	-	-	-	-	-	-	-	-	3	5	8
	F	-	-	-	-	-	-	-	-	-	1	3	4
SB6	M	-	-	-	-	-	-	-	-	-	-	3	3
	F	-	-	-	-	-	-	-	-	-	-	3	3
SB7	M	-	-	-	2	2	3	2	2	2	2	13	28
	F	-	-	-	3	3	2	4	2	3	2	15	34
SB8	M	-	-	-	-	-	-	-	-	-	-	-	-
	F	-	-	-	-	-	-	-	-	-	-	-	-
SB9	M	-	-	-	-	-	-	-	-	-	-	-	-
	F	-	-	-	-	-	-	-	-	-	-	-	-
SD1	M	-	-	-	2	3	3	3	1	2	2	6	22
	F	-	-	-	3	2	3	4	3	2	3	9	29
SD2	M	-	-	-	-	-	-	-	-	-	-	-	-
	F	-	-	-	-	-	-	-	-	-	-	-	-
SCa	M	-	1	-	-	-	-	-	-	-	-	-	1
	F	-	1	-	-	-	-	-	-	-	-	-	1
BS	M	77	39	-	-	-	-	-	-	-	-	-	116
	F	76	38	-	-	-	-	-	-	-	-	-	114

Table S6. Distribution and numbers of different sensilla on the antennomeres I–XI in *Agriotes obscurus* (Ag22, Ag25). M, male; F, female; SC1 and SC2, sensilla chaetica subtypes 1 and 2, respectively; ST, sensilla trichodea; SB1–9, sensilla basiconica subtypes 1–9; SD1–2, dome-shaped sensilla subtypes 1–2; SCa, sensilla campaniformia; BS, Böhm sensilla.

Sensilla	Sex	Antennomere											
		I	II	III	IV	V	VI	VII	VIII	IX	X	XI	Total
SC1	M	169	80	99	123	131	137	145	154	163	178	184	1563
	F	182	85	107	134	158	149	178	182	195	214	277	1861
SC2	M	6	5	4	5	6	6	6	7	7	7	13	72
	F	8	4	6	6	7	9	8	7	7	7	15	84
ST	M	-	-	-	14	25	52	66	78	83	107	201	626
	F	-	-	-	13	16	17	15	21	19	16	22	139
SB1	M	-	-	-	21	35	43	46	51	57	60	64	377
	F	-	-	-	49	60	68	72	70	69	77	32	497
SB2	M	-	-	-	15	28	20	25	26	32	36	42	224
	F	-	-	-	17	15	20	21	24	38	45	69	249
SB3	M	-	-	-	-	-	-	-	-	-	1	5	6
	F	-	-	-	-	-	-	-	-	-	2	8	10
SB4	M	-	-	-	-	-	-	1	3	5	4	5	18
	F	-	-	-	-	-	-	2	3	6	5	8	24
SB5	M	-	-	-	-	-	-	-	-	-	2	5	7
	F	-	-	-	-	-	-	-	-	-	1	4	5
SB6	M	-	-	-	-	-	-	-	-	-	-	3	3
	F	-	-	-	-	-	-	-	-	-	-	6	6
SB7	M	-	-	-	2	2	3	2	2	2	2	15	30
	F	-	-	-	3	3	2	3	3	3	2	14	33
SB8	M	-	-	-	-	-	-	-	-	-	-	-	-
	F	-	-	-	-	-	-	-	-	-	-	-	-
SB9	M	-	-	-	-	-	-	-	-	-	-	-	-
	F	-	-	-	-	-	-	-	-	-	-	-	-
SD1	M	-	-	-	2	3	3	3	2	4	3	10	30
	F	-	-	-	3	2	2	4	3	3	4	11	32
SD2	M	-	-	-	-	-	-	-	-	-	-	-	-
	F	-	-	-	-	-	-	-	-	-	-	-	-
SCa	M	-	1	-	-	-	-	-	-	-	-	-	1
	F	-	1	-	-	-	-	-	-	-	-	-	1
BS	M	75	38	-	-	-	-	-	-	-	-	-	113
	F	73	39	-	-	-	-	-	-	-	-	-	112

Table S7. Distribution and numbers of different sensilla on the antennomeres I–XI in *Agriotes paludum* (Ag41, Ag42). M, male; F, female; SC1 and SC2, sensilla chaetica subtypes 1 and 2, respectively; ST, sensilla trichodea; SB1–9, sensilla basiconica subtypes 1–9; SD1–2, dome-shaped sensilla subtypes 1–2; SCa, sensilla campaniformia; BS, Böhm sensilla.

**Sensilla**	**Sex**	**Antennomere**											
		**I**	**II**	**III**	**IV**	**V**	**VI**	**VII**	**VIII**	**IX**	**X**	**XI**	**Total**
SC1	M	117	69	65	101	108	127	102	117	104	112	155	1177
	F	129	72	68	114	116	133	139	158	165	177	204	1475
SC2	M	7	5	6	6	6	7	8	7	7	8	12	79
	F	8	5	7	7	8	8	7	7	7	8	16	88
ST	M	-	-	-	11	23	47	59	68	71	112	144	535
	F	-	-	-	9	13	15	15	18	16	19	14	119
SB1	M	-	-	-	28	37	42	46	51	48	50	54	356
	F	-	-	-	41	46	65	70	74	76	79	48	499
SB2	M	-	-	-	19	28	30	34	29	34	28	35	237
	F	-	-	-	21	25	34	39	33	41	47	54	294
SB3	M	-	-	-	-	-	-	-	-	-	-	6	6
	F	-	-	-	-	-	-	-	-	-	2	8	10
SB4	M	-	-	-	-	-	-	1	2	5	4	7	19
	F	-	-	-	-	-	-	2	4	6	6	8	26
SB5	M	-	-	-	-	-	-	-	-	-	-	4	4
	F	-	-	-	-	-	-	-	-	-	-	3	3
SB6	M	-	-	-	-	-	-	-	-	-	-	2	2
	F	-	-	-	-	-	-	-	-	-	-	3	3
SB7	M	-	-	-	1	2	2	2	3	3	2	10	25
	F	-	-	-	3	3	4	3	2	3	4	12	34
SB8	M	-	-	-	-	-	-	-	-	-	-	-	-
	F	-	-	-	-	-	-	-	-	-	-	-	-
SB9	M	-	-	-	-	-	-	-	-	-	-	-	-
	F	-	-	-	-	-	-	-	-	-	-	-	-
SD1	M	-	-	-	1	2	2	1	3	3	2	7	21
	F	-	-	-	1	3	2	2	2	3	3	6	22
SD2	M	-	-	-	-	-	-	-	-	-	-	-	-
	F	-	-	-	-	-	-	-	-	-	-	-	-
SCa	M	-	1	-	-	-	-	-	-	-	-	-	1
	F	-	1	-	-	-	-	-	-	-	-	-	1
BS	M	76	38	-	-	-	-	-	-	-	-	-	114
	F	73	41	-	-	-	-	-	-	-	-	-	114

Table S8. Distribution and numbers of different sensilla on the antennomeres I–XI in *Agriotes pilosellus* (Ag27, Ag29). M, male; F, female; SC1 and SC2, sensilla chaetica subtypes 1 and 2, respectively; ST, sensilla trichodea; SB1–9, sensilla basiconica subtypes 1–9; SD1–2, dome-shaped sensilla subtypes 1–2; SCa, sensilla campaniformia; BS, Böhm sensilla.

Sensilla	Sex	Antennomere											
		I	II	III	IV	V	VI	VII	VIII	IX	X	XI	Total
SC1	M	197	65	72	88	90	96	97	109	112	128	140	1194
	F	109	79	84	95	113	129	133	154	163	152	176	1387
SC2	M	6	4	5	4	4	5	5	5	7	7	8	60
	F	7	4	6	5	6	6	6	6	8	7	9	70
ST	M	-	-	-	19	25	56	69	75	79	110	129	562
	F	-	-	-	11	14	13	10	16	11	13	18	106
SB1	M	-	-	-	46	57	59	68	72	69	87	90	548
	F	-	-	-	55	60	67	81	88	90	96	105	642
SB2	M	-	-	-	31	33	35	43	46	51	45	54	338
	F	-	-	-	28	35	39	50	52	52	49	70	375
SB3	M	-	-	-	-	-	-	-	-	-	-	4	4
	F	-	-	-	-	-	-	-	-	-	1	10	11
SB4	M	-	-	-	-	-	-	2	2	4	3	5	16
	F	-	-	-	-	-	-	3	3	5	4	7	22
SB5	M	-	-	-	-	-	-	-	-	-	1	8	9
	F	-	-	-	-	-	-	-	-	-	2	5	7
SB6	M	-	-	-	-	-	-	-	-	-	-	4	4
	F	-	-	-	-	-	-	-	-	-	-	5	5
SB7	M	-	-	-	4	3	2	2	3	2	2	15	33
	F	-	-	-	3	2	3	2	4	2	2	17	35
SB8	M	-	-	-	-	-	-	-	-	-	-	-	-
	F	-	-	-	-	-	-	-	-	-	-	-	-
SB9	M	-	-	-	-	-	-	-	-	2	3	1	6
	F	-	-	-	-	-	-	-	-	2	1	4	7
SD1	M	-	-	-	3	3	1	2	3	2	2	6	22
	F	-	-	-	2	2	2	3	2	1	2	5	19
SD2	M	-	-	-	-	-	-	-	-	-	-	-	-
	F	-	-	-	-	-	-	-	-	-	-	-	-
SCa	M	-	1	-	-	-	-	-	-	-	-	-	1
	F	-	1	-	-	-	-	-	-	-	-	-	1
BS	M	78	40	-	-	-	-	-	-	-	-	-	118
	F	74	38	-	-	-	-	-	-	-	-	-	112

Table S9. Distribution and numbers of different sensilla on the antennomeres I–XI in *Agriotes rufipalpis* (Ag30, Ag31). M, male; F, female; SC1 and SC2, sensilla chaetica subtypes 1 and 2, respectively; ST, sensilla trichodea; SB1–9, sensilla basiconica subtypes 1–9; SD1–2, dome-shaped sensilla subtypes 1–2; SCa, sensilla campaniformia; BS, Böhm sensilla.

**Sensilla**	**Sex**	**Antennomere**											
		**I**	**II**	**III**	**IV**	**V**	**VI**	**VII**	**VIII**	**IX**	**X**	**XI**	**Total**
SC1	M	118	62	54	87	89	95	108	110	127	131	151	1132
	F	124	75	70	92	97	104	115	133	142	148	186	1286
SC2	M	7	4	5	4	5	6	6	6	6	7	12	68
	F	7	5	6	5	6	7	7	6	7	7	13	76
ST	M	-	-	-	7	10	23	48	56	69	85	110	408
	F	-	-	-	10	8	11	13	17	15	12	11	97
SB1	M	-	-	-	24	46	52	58	49	62	68	60	419
	F	-	-	-	55	78	81	77	65	68	59	39	522
SB2	M	-	-	-	20	23	21	27	24	29	35	28	207
	F	-	-	-	26	32	29	23	30	48	53	67	308
SB3	M	-	-	-	-	-	-	-	-	-	1	6	7
	F	-	-	-	-	-	-	-	-	-	2	9	11
SB4	M	-	-	-	-	-	-	-	3	5	5	6	19
	F	-	-	-	-	-	-	3	4	6	5	8	26
SB5	M	-	-	-	-	-	-	-	-	-	2	3	5
	F	-	-	-	-	-	-	-	-	-	1	3	4
SB6	M	-	-	-	-	-	-	-	-	-	-	2	2
	F	-	-	-	-	-	-	-	-	-	-	4	4
SB7	M	-	-	-	4	2	2	3	5	4	3	11	34
	F	-	-	-	5	8	4	4	4	4	3	9	41
SB8	M	-	-	-	-	-	-	-	-	-	-	-	-
	F	-	-	-	-	-	-	-	-	-	-	-	-
SB9	M	-	-	-	-	-	-	-	-	-	-	-	-
	F	-	-	-	-	-	-	-	-	-	-	-	-
SD1	M	-	-	-	3	1	1	3	2	2	2	11	25
	F	-	-	-	2	2	1	2	2	1	2	9	21
SD2	M	-	-	-	-	-	-	-	-	-	-	-	-
	F	-	-	-	-	-	-	-	-	-	-	-	-
SCa	M	-	1	-	-	-	-	-	-	-	-	-	1
	F	-	1	-	-	-	-	-	-	-	-	-	1
BS	M	75	39	-	-	-	-	-	-	-	-	-	114
	F	74	39	-	-	-	-	-	-	-	-	-	113

Table S10. Distribution and numbers of different sensilla on the antennomeres I–XI in *Agriotes sputator* (Ag33, Ag35). M, male; F, female; SC1 and SC2, sensilla chaetica subtypes 1 and 2, respectively; ST, sensilla trichodea; SB1–9, sensilla basiconica subtypes 1–9; SD1–2, dome-shaped sensilla subtypes 1–2; SCa, sensilla campaniformia; BS, Böhm sensilla.

Sensilla	Sex	Antennomere											
		I	II	III	IV	V	VI	VII	VIII	IX	X	XI	Total
SC1	M	132	72	67	98	92	95	104	117	119	148	161	1205
	F	146	74	72	101	99	102	108	132	128	202	237	1401
SC2	M	6	3	6	6	6	6	6	6	6	7	10	68
	F	6	4	8	7	7	7	6	7	7	7	13	79
ST	M	-	-	-	9	13	33	52	66	75	89	153	490
	F	-	-	-	7	9	9	14	15	11	13	10	88
SB1	M	-	-	-	30	60	55	52	56	54	65	46	418
	F	-	-	-	34	67	58	60	62	57	73	64	475
SB2	M	-	-	-	17	24	22	25	29	36	37	39	229
	F	-	-	-	13	28	24	32	29	38	44	58	266
SB3	M	-	-	-	-	-	-	-	-	-	2	3	5
	F	-	-	-	-	-	-	-	-	-	2	8	10
SB4	M	-	-	-	-	-	-	-	2	6	4	7	19
	F	-	-	-	-	-	-	2	3	7	6	7	25
SB5	M	-	-	-	-	-	-	-	-	-	2	6	8
	F	-	-	-	-	-	-	-	-	-	-	4	4
SB6	M	-	-	-	-	-	-	-	-	-	-	3	3
	F	-	-	-	-	-	-	-	-	-	-	5	5
SB7	M	-	-	-	2	2	2	4	3	4	2	5	24
	F	-	-	-	3	3	-	3	2	2	3	7	23
SB8	M	-	-	-	-	-	-	-	-	-	-	-	-
	F	-	-	-	-	-	-	-	-	-	-	-	-
SB9	M	-	-	-	-	-	-	-	-	-	-	-	-
	F	-	-	-	-	-	-	-	-	-	-	-	-
SD1	M	-	-	-	2	2	2	1	3	2	2	6	20
	F	-	-	-	2	2	1	1	2	1	3	7	19
SD2	M	-	-	-	-	-	-	-	-	-	-	-	-
	F	-	-	-	-	-	-	-	-	-	-	-	-
SCa	M	-	1	-	-	-	-	-	-	-	-	-	1
	F	-	1	-	-	-	-	-	-	-	-	-	1
BS	M	74	39	-	-	-	-	-	-	-	-	-	113
	F	71	40	-	-	-	-	-	-	-	-	-	111

Table S11. Distribution and numbers of different sensilla on the antennomeres I–XI in *Agriotes ustulatus* (Ag37, Ag39). M, male; F, female; SC1 and SC2, sensilla chaetica subtypes 1 and 2, respectively; ST, sensilla trichodea; SB1–9, sensilla basiconica subtypes 1–9; SD1–2, dome-shaped sensilla subtypes 1–2; SCa, sensilla campaniformia; BS, Böhm sensilla.

Sensilla	Sex	Antennomere											
		I	II	III	IV	V	VI	VII	VIII	IX	X	XI	Total
SC1	M	85	52	31	56	67	65	78	84	82	96	109	805
	F	94	60	24	71	78	82	81	105	117	119	138	969
SC2	M	5	5	6	8	7	7	7	6	7	6	11	75
	F	6	5	7	7	7	8	8	8	8	8	16	88
ST	M	-	-	-	15	23	54	62	77	76	105	129	541
	F	-	-	-	13	17	16	18	24	21	12	24	145
SB1	M	-	-	-	12	29	33	39	47	62	49	74	345
	F	-	-	-	20	33	37	42	56	74	70	85	417
SB2	M	-	-	-	9	17	25	32	26	32	34	39	214
	F	-	-	-	12	22	28	36	28	35	42	64	267
SB3	M	-	-	-	-	-	-	-	-	-	1	7	8
	F	-	-	-	-	-	-	-	-	-	1	9	10
SB4	M	-	-	-	-	-	-	-	3	5	3	6	17
	F	-	-	-	-	-	-	1	3	5	6	7	22
SB5	M	-	-	-	-	-	-	-	-	-	3	6	9
	F	-	-	-	-	-	-	-	-	-	1	5	6
SB6	M	-	-	-	-	-	-	-	-	-	-	4	4
	F	-	-	-	-	-	-	-	-	-	-	7	7
SB7	M	-	-	-	2	2	3	1	3	5	2	3	21
	F	-	-	-	3	3	4	3	5	7	2	5	32
SB8	M	-	-	-	-	-	-	-	-	-	-	-	-
	F	-	-	-	-	-	-	-	-	-	-	-	-
SB9	M	-	-	-	-	-	-	-	-	-	-	-	-
	F	-	-	-	-	-	-	-	-	-	-	-	-
SD1	M	-	-	-	3	2	-	2	2	2	2	8	21
	F	-	-	-	3	2	1	3	2	2	2	5	20
SD2	M	-	-	-	-	-	-	-	-	-	-	-	-
	F	-	-	-	-	-	-	-	-	-	-	-	-
SCa	M	-	1	-	-	-	-	-	-	-	-	-	1
	F	-	1	-	-	-	-	-	-	-	-	-	1
BS	M	76	37	-	-	-	-	-	-	-	-	-	113
	F	75	39	-	-	-	-	-	-	-	-	-	114
